# Integrating ecosystem services considerations within a GIS-based habitat suitability index for oyster restoration

**DOI:** 10.1371/journal.pone.0210936

**Published:** 2019-01-25

**Authors:** Seth J. Theuerkauf, David B. Eggleston, Brandon J. Puckett

**Affiliations:** 1 North Carolina State University, Department of Marine, Earth and Atmospheric Sciences, Center for Marine Sciences and Technology, Morehead City, North Carolina, United States of America; 2 North Carolina Coastal Reserve and National Estuarine Research Reserve, Beaufort, North Carolina, United States of America; University of Alabama, UNITED STATES

## Abstract

Geospatial habitat suitability index (HSI) models have emerged as powerful tools that integrate pertinent spatial information to guide habitat restoration efforts, but have rarely accounted for spatial variation in ecosystem service provision. In this study, we utilized satellite-derived chlorophyll *a* concentrations for Pamlico Sound, North Carolina, USA in conjunction with data on water flow velocities and dissolved oxygen concentrations to identify potential restoration locations that would maximize the oyster reef-associated ecosystem service of water filtration. We integrated these novel factors associated with oyster water filtration ecosystem services within an existing, ‘Metapopulation Persistence’ focused GIS-based, HSI model containing biophysical (e.g., salinity, oyster larval connectivity) and logistical (e.g., distance to nearest restoration material stockpile site) factors to identify suitable locations for oyster restoration that maximize long-term persistence of restored oyster populations and water filtration ecosystem service provision. Furthermore, we compared the ‘Water Filtration’ optimized HSI with the HSI optimized for ‘Metapopulation Persistence,’ as well as a hybrid model that optimized for both water filtration and metapopulation persistence. Optimal restoration locations (i.e., locations corresponding to the top 1% of suitability scores) were identified that were consistent among the three HSI scenarios (i.e., “win-win” locations), as well as optimal locations unique to a given HSI scenario (i.e., “tradeoff” locations). The modeling framework utilized in this study can provide guidance to restoration practitioners to maximize the cost-efficiency and ecosystem services value of habitat restoration efforts. Furthermore, the functional relationships between oyster water filtration and chlorophyll *a* concentrations, water flow velocities, and dissolved oxygen applied in this study can guide field- and lab-testing of hypotheses related to optimal conditions for oyster reef restoration to maximize water quality enhancement benefits.

## Introduction

Recovery of ecosystem services is often cited as a principal motivation for habitat restoration activities [1]. Yet, the quantity and quality of ecosystem services provided by restored habitats can vary greatly in space and time and are often mediated by restored habitat quality [2, 3, 4]. Geospatial habitat suitability indices (hereafter ‘HSI’) have emerged as powerful, spatially explicit decision support tools to guide habitat restoration of areas with the highest probable habitat quality [5]. HSIs are commonly generated through application of wildlife-habitat relationships with relevant geospatial environmental data within a Geographic Information System (GIS) to develop a composite HSI score with a range of 0 to 1, representing unsuitable (0) to optimal (1) habitat [6]. Previous efforts have sought to map and quantify spatial variation in ecosystem services provided by existing habitats (e.g., [7]), however, relatively few efforts have attempted to assess where habitat restoration might provide enhanced levels of ecosystem service provision relative to other locations. Given the need and desire to maximize provision of ecosystem services associated with habitat restoration efforts, as well as the often significant associated costs of restoration (e.g., US$10,000 per ha per cm of substrate material for oyster habitat restoration in Chesapeake Bay [8]), there is a need for a geospatial modeling framework to inform where habitat restoration efforts might be most successful and yield the greatest ecosystem services benefit.

Oyster reefs provide important ecosystem services within estuaries, including water filtration, sediment stabilization, and provision of essential fish habitat [9]. Despite their recognized value, native oyster populations worldwide are at ~10–15% of their historic levels due to a combination of overfishing, habitat destruction, and disease [10]. The global loss of native oyster reefs has prompted the establishment of large-scale oyster restoration programs to restore oyster populations to recover these lost ecosystem services. Recent research into the water quality enhancement benefits associated with oyster reef restoration (e.g., removal of phytoplankton biomass from the water column and enhancement of denitrification rates; [11, 12]), coupled with the rising cost of infrastructural means of reducing nutrient pollution (e.g., improvements to wastewater treatment facilities) has generated considerable interest in the role of strategic oyster restoration as a cost-effective means to improve water quality and meet nutrient reduction mandates [13]. Restoration of oyster reefs to maximize potential water quality benefits requires, in part, a spatial understanding of where the ecosystem service of oyster filtration would be greatest.

Geospatial HSI models that integrate relevant environmental, biological, and logistical factors are useful tools to identify optimal sites for habitat restoration within the broader landscape or seascape of interest [5]. Multiple HSI models have been developed to guide aquaculture, fishery production, and restoration of oyster species [14, 15, 16, 17]. These models have incorporated a range of abiotic and biotic factors of relevance to oyster restoration, such as salinity, bottom type, and water depth. The combinations of factors incorporated into these waterbody-specific HSIs have varied depending upon both data availability and relevance in determining habitat suitability for a given system. To date, none of the published oyster restoration HSIs has directly incorporated factors pertinent to, and for purposes of, identification of suitable restoration locations that would maximize ecosystem service provision.

The present study extends an HSI originally developed by Puckett et al. [18] to guide oyster habitat restoration activities in Pamlico Sound, North Carolina, USA by incorporating ecosystem services-related factors to identify oyster reef restoration locations that maximize provision of water filtration ecosystem services [18]. The original HSI developed by Puckett et al. [18] incorporates biophysical (e.g., salinity, oyster larval connectivity) and logistical (e.g., distance to nearest restoration material stockpile site) factors essential to restoration contributing to long-term oyster metapopulation persistence (hereafter ‘Metapopulation Persistence HSI’) [18]. Specifically, the ‘Metapopulation Persistence HSI’ is optimized to identify restoration locations that maximize the likelihood of persistence of an individual restored reef and maximize an individual restored reef’s larval connectivity to the overall oyster metapopulation of Pamlico Sound (i.e., larval import and export are weighted heavily), thereby improving likelihood of metapopulation persistence. In the present study, we utilize satellite-derived chlorophyll *a* concentrations for Pamlico Sound in conjunction with data on water flow velocities, dissolved oxygen concentrations, and varying combinations of factors considered within the original ‘Metapopulation Persistence HSI’ to generate two additional HSI scenarios: one focused primarily on identifying restoration locations that would maximize likelihood of persistence of an individual restored reef and oyster water filtration ecosystem services (hereafter, ‘Water Filtration HSI’), and another that balanced long-term oyster population persistence criteria with oyster water filtration ecosystem services considerations (hereafter, ‘Water Filtration & Metapopulation Persistence HSI’). We subsequently compared both suitability patterns and optimal locations (i.e., locations corresponding to the top 1% of HSI values) identified within and between all three HSI model scenarios to determine optimal restoration locations that were consistent among scenarios (i.e., “win-win” locations), as well as optimal locations unique to each scenario (i.e., “tradeoff” locations). We also evaluated the sensitivity of each HSI scenario to its respective parameterization to determine which factors are major drivers of suitability within a given HSI. The conceptual framework utilized in this study, wherein restoration goal-specific HSIs (e.g., maximizing long-term population persistence, maximizing ecosystem service provision) were developed and “win-win” versus “tradeoff” locations were identified can broadly inform development of similar restoration goal-specific HSI models in other systems. Moreover, the functional relationships between oyster water filtration and chlorophyll *a* concentrations, water flow velocities, and dissolved oxygen developed in this study can guide field- and lab-testing of hypotheses related to optimal conditions for oyster reef restoration to maximize water quality enhancement benefits.

## Methods

### Study system

**Fig 1:**
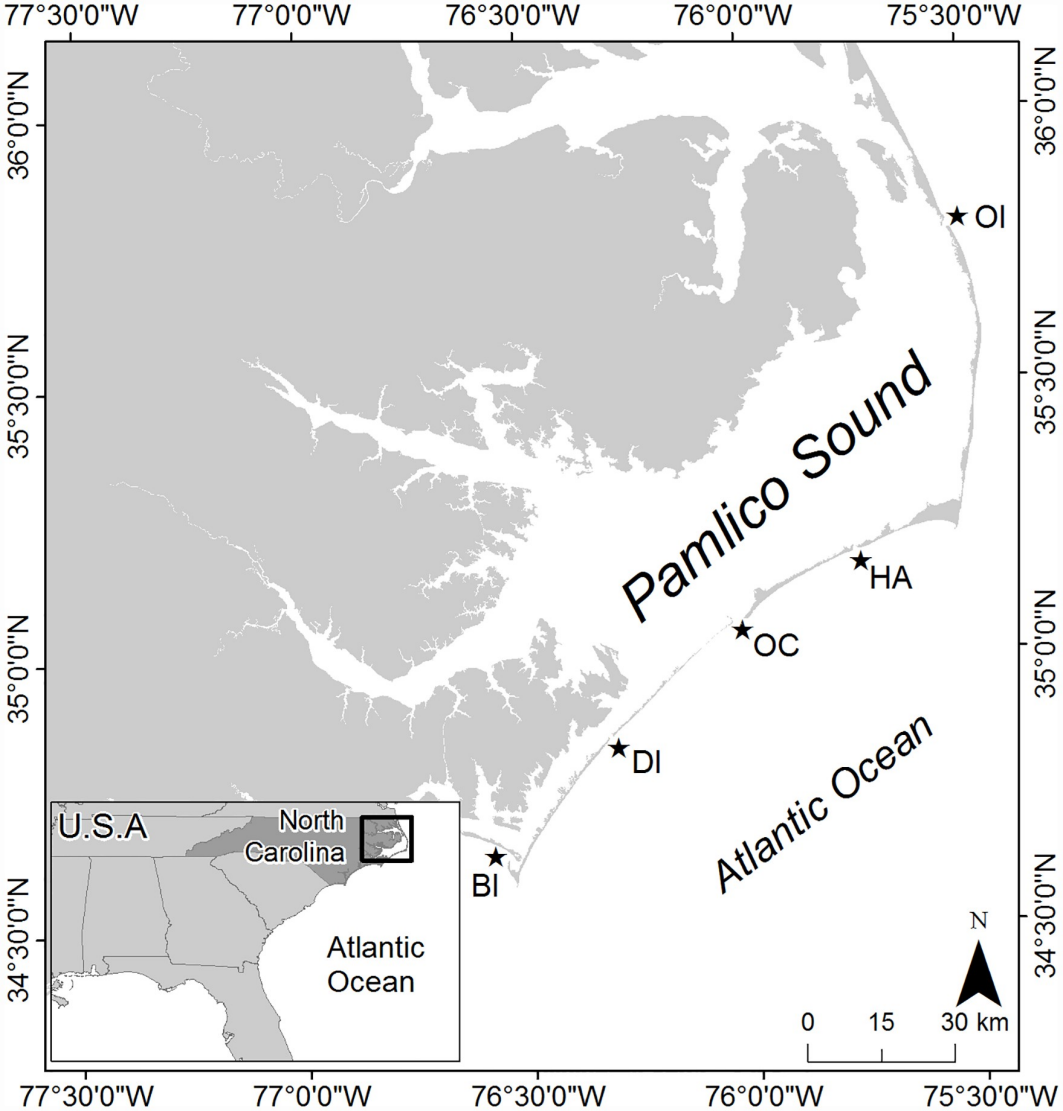
Map showing the location of the study area, Pamlico Sound, within the Albemarle-Pamlico Estuarine System (APES); stars denote oceanic inlets (OI = Oregon Inlet, HA = Hatteras Inlet, OC = Ocracoke Inlet, DI = Drum Inlet, BI = Bardens Inlet).

The Albemarle-Pamlico Estuarine System (APES) in North Carolina, USA is the largest lagoonal estuary in the United States (~6,600 km^2^), and is bounded by a barrier island chain that limits exchange with the coastal ocean to five relatively small inlets (~1 km wide; Fig 1 [19, 20]). Pamlico Sound, the largest component of the APES (~120 x 40 km), is relatively shallow with a mean depth of ~4.5 m and a maximum depth of 7.5 m [19]. The shallow nature of this wind-driven estuary coupled with limited oceanic exchange yields a relatively long water residence time and negligible vertical stratification of the water column (i.e., high degree of vertical mixing of the water column [21]. Previous research has indicated that phytoplankton community structure and biomass is consistent vertically throughout the water column in the well-mixed portion of North Carolina estuaries [22], congruent with patterns identified in previous studies of well-mixed estuaries, such as San Francisco Bay, California, USA and Bay of Brest, France [23, 24].

Subtidal oyster reefs, a once prevalent benthic habitat in Pamlico Sound, are believed to occupy ~1–10% of their historical footprint [10, 18]. Multiple subtidal oyster habitat restoration methods are ongoing, including: (1) cultch planting, the deployment of a thin veneer of oyster shell or other settlement substrate to replace shell removed through commercial harvest, and (2) no-harvest sanctuaries, the designation of areas protected from harvest within which large, high-relief artificial reefs are constructed to provide settlement substrate [25, 26, 27]. The present study provides spatial guidance to inform both forms of subtidal oyster reef restoration, but contains recommendations most pertinent to restoration of ‘no-harvest sanctuaries’ by including factors such as depth requirements to ensure safe navigational clearance when restoring high-relief reefs with large material (e.g., granite rock, reef balls).

### Original HSI model characteristics

This study extends a previous GIS-based habitat suitability index (HSI) developed for restoration of eastern oysters (*Crassostrea virginica*) in Pamlico Sound, North Carolina, USA [18]. The original model was developed by: 1) convening stakeholder meetings to identify model input parameters and their relative importance to oyster restoration, 2) using a GIS-based modeling approach to integrate 17 physical, biological, and logistical parameters to identify optimal locations for habitat restoration that maximize persistence of oyster populations on restored reefs (i.e., ‘Metapopulation Persistence HSI’; S1 Table), and 3) conducting sensitivity analysis and model validation analyses to assess model performance.

Using ArcMap 10.3 [28], a grid was developed consisting of 5,987 1 km x 1 km (1 km^2^) grid cells covering the waters of Pamlico Sound [18]. Using expert stakeholder input (i.e., academics, non-governmental organizations, and state resource managers engaged in oyster restoration), 17 GIS layers were projected onto the grid of Pamlico Sound such that each cell contained a “value” for each layer. Layers were partitioned into two categories: (1) “threshold” layers—those assigned values based on thresholds and weights relevant to suitability (S1 Fig), and (2) “exclusion” layers—binary, 0 or 1 layers used to exclude unsuitable sites (S2 Fig). Threshold values and weights, as well as exclusion layer values were determined through literature reviews, stakeholder input, and regulatory statutes (Table 1, S1 Table). A complete list of all threshold and exclusion layers, and their associated details can be found in S1 Table.

**Table 1:** Threshold layers and associated weights utilized to compute suitability in the three HSI scenarios. Weights were applied to each layer, and the assigned weight corresponds to the relative importance of each threshold layer to siting oyster restoration efforts in a given HSI scenario. The assigned weights of all threshold layers sum to 100%.

Threshold Layers	Water Filtration HSI	Water Filtration & Metapopulation Persistence HSI	Metapopulation Persistence HSI
Salinity	10%	15%	23%
Sanctuary Reef Larval Export	8%	11%	20%
Sanctuary Reef Larval Import	**-**	6%	15%
Dissolved Oxygen	19%	12%	11%
Cultch Reef Larval Import	**-**	2%	7%
Natural Reef Larval Import	**-**	2%	7%
Cultch Reef Larval Export	3%	5%	5%
Natural Reef Larval Export	3%	5%	5%
Material Stockpile Site Proximity	1.5%	4%	4%
Boat Ramp Proximity	1.5%	4%	3%
Chlorophyll a (Mean)	28%	17%	**-**
Chlorophyll a (Variation)	10%	7%	**-**
Flow (Mean + Variation)	16%	10%	**-**

**Fig 2:**
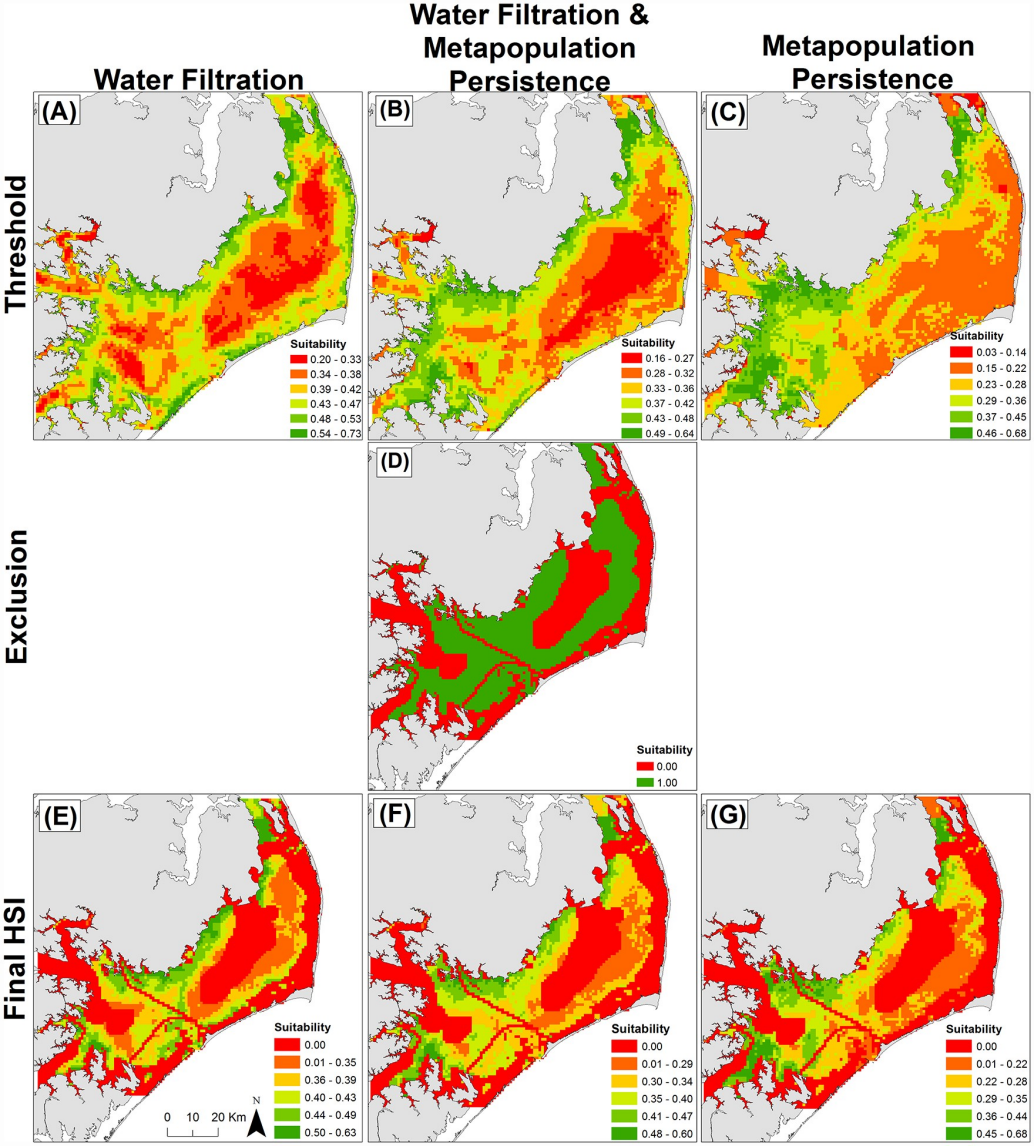
Habitat suitability based on: (A-C) aggregated threshold layers, (D) aggregated exclusion layers, (E-G) aggregated exclusion and threshold layers combined for the three model scenarios: ‘Water Filtration,’ ‘Water Filtration & Metapopulation Persistence,’ and ‘Metapopulation Persistence’. Suitability in panels A-C and E-G is continuous, while suitability in panel D is binary. Suitability increases from low (red) to high (green) HSI. Panels C, D, and G adapted from Puckett et al. [18].

Using the GIS-raster calculator, the suitability of each cell (*S*_*j*_) for siting restored oyster reefs within the ‘Metapopulation Persistence HSI’ was calculated in a two-step process as:

$$C_{\;j}\;=\;\sum_{x\;=\;1}^{10}(L_{xj}\;∙W_{x})$$

$$S_{j}\;=\;C_{j}∙E_{j,}$$

where *C*_*j*_ is the cumulative value of cell *j* calculated as the product of the threshold value *L* of cell *j* in threshold layer *x* and the weight *W* of layer *x* summed across all 10 threshold layers, and *E*_*j*_ is the binary (0 or 1) score for cell *j* based on the product of all 7 binary exclusion layers. On a scale of 0 to 1, cell suitability scores for restored oyster reefs were ranked from lowest (least suitable) to highest (most suitable). Output from the original ‘Metapopulation Persistence HSI’ is provided in Fig 2C and 2G. The present study extends this original modeling effort by integrating novel spatial layers related to water quality enhancement ecosystem services within a ‘Water Filtration HSI’, and a hybrid HSI optimized for ‘Water Filtration & Metapopulation Persistence.’

### Water filtration ecosystem services layer development

To develop a ‘Water Filtration HSI’, we focused on relevant input parameters for which: 1) spatially-explicit datasets were available for our study system, and 2) functional relationships between these abiotic and biotic parameters and suitability for oyster filtration could be inferred from the literature. Based on these criteria, we included: chlorophyll *a* concentrations, water flow velocities, and dissolved oxygen (DO) concentrations. We subsequently applied suitability functions based on literature-inferred relationships to available spatial datasets and integrated these layers along with other layers relevant to each HSI scenario (i.e., ‘Water Filtration,’ ‘Water Filtration & Metapopulation Persistence,’ and ‘Metapopulation Persistence’; see ‘Methods, *HSI Integration’* below).

Chlorophyll *a* concentrations serve as a surrogate variable for phytoplankton biomass (i.e., food availability). Recent oyster reef growth modeling efforts have described the importance of selecting restoration locations with greatest food availability in determining long-term reef survival [29]. Water flow velocity is an important factor regulating food delivery and oyster filtration. Increasing water flow velocities across reefs to 15 cm s^-1^ increases the rate of food delivery [30, 31], however exceedance of 15 cm s^-1^ can result in sediment resuspension or cessation of oyster filtration, yielding no net reduction in seston concentrations [11, 30, 32]. Areas of high chlorophyll *a* concentrations have been associated with areas of low benthic DO concentrations (i.e., hypoxic or anoxic “dead zones”) that can be lethal to oysters; therefore, DO concentrations are a critical additional consideration [33].

**Chlorophyll *a* concentration** (μg chl *a* l^-1^) information was derived from monthly-averaged, 300-m spatial resolution satellite images (i.e., average of all satellite passes within a given month for the period of January 2003 through December 2011) captured by the European Space Agency’s Environmental Satellite 1 (EnviSat-1) Medium Resolution Imaging Spectrometer (MERIS). All chlorophyll *a* data products were provided in a pre-processed form by the United States Environmental Protection Agency (Blake Schaeffer, personal communication). Keith (2014) performed validation of MERIS-derived chlorophyll *a* concentrations for Pamlico Sound and tributaries using *in situ*-measured chlorophyll *a* concentrations, and identified a statistically-significant ~1:1 relationship between the two data sources [34]. MERIS imagery is highly suitable for estimation of chlorophyll *a* concentrations given its narrow spectral bands with a high signal to noise ratio [35]. To determine which month across the nine-year data series represented the average chlorophyll *a* maximum when phytoplankton biomass within Pamlico Sound overall is maximized to aid identification of locations where maximum water filtration benefits of restoration would be realized, we averaged chlorophyll *a* concentrations across all pixels within a grid for a given sampling period across years within a given month (e.g., averaged across January 2003 through 2011). From this analysis, September, which corresponds with the fall phytoplankton bloom in Pamlico Sound, was determined to be the month of maximum average chlorophyll *a* concentration for the period of 2003 through 2011 (S2 Table). This timing also corresponds with elevated water temperatures throughout the system [19, 20, 21] that would support maximum oyster filtration rates [14].

**Fig 3:**
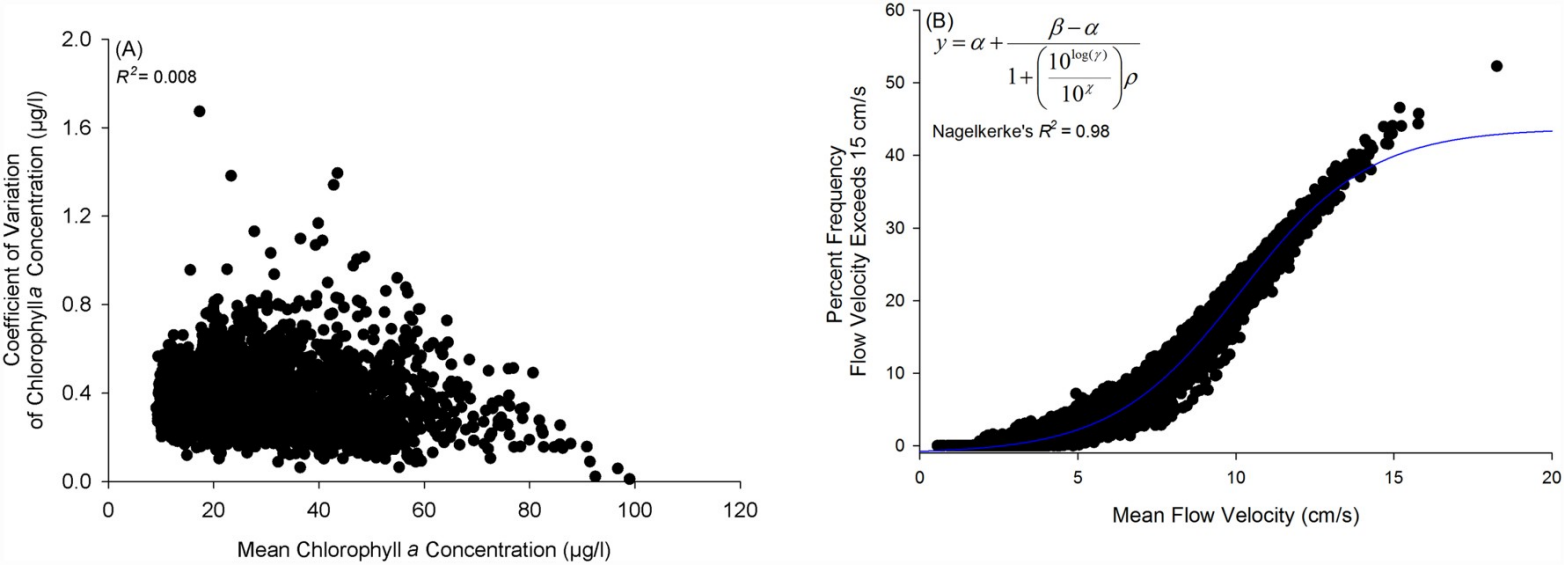
Relationships between: (A) mean chlorophyll *a* concentration and coefficient of variation, and (B) mean flow velocity (cm/s) and percent frequency flow velocity exceeds 15 cm/s. A description of the analysis methods used to identify these relationships can be found in ‘*Methods*, *Water Filtration Ecosystem Services Layer Development*’.

We calculated the: 1) mean and 2) coefficient of variation for chlorophyll *a* for each cell within the model grid using data from September of 2003 through 2011. The coefficient of variation (c_v_ = σ μ^-1^) provides a standardized statistic to compare the degree of variation amongst data points irrespective of mean values. We evaluated the form of the relationship between mean chlorophyll *a* concentration and the coefficient of variation using the local polynomial regression fitting (loess) function in R [36]. Based on the relationships from the loess fits, mean chlorophyll *a* concentration and the variance to mean ratio appeared to be uncorrelated, which was further confirmed by simple linear regression (Fig 3A, *R*^*2*^ = 0.008). Thus, we developed separate spatial layers to represent: 1) mean chlorophyll *a* concentration and 2) coefficient of variation of chlorophyll *a*.

**Fig 4:**
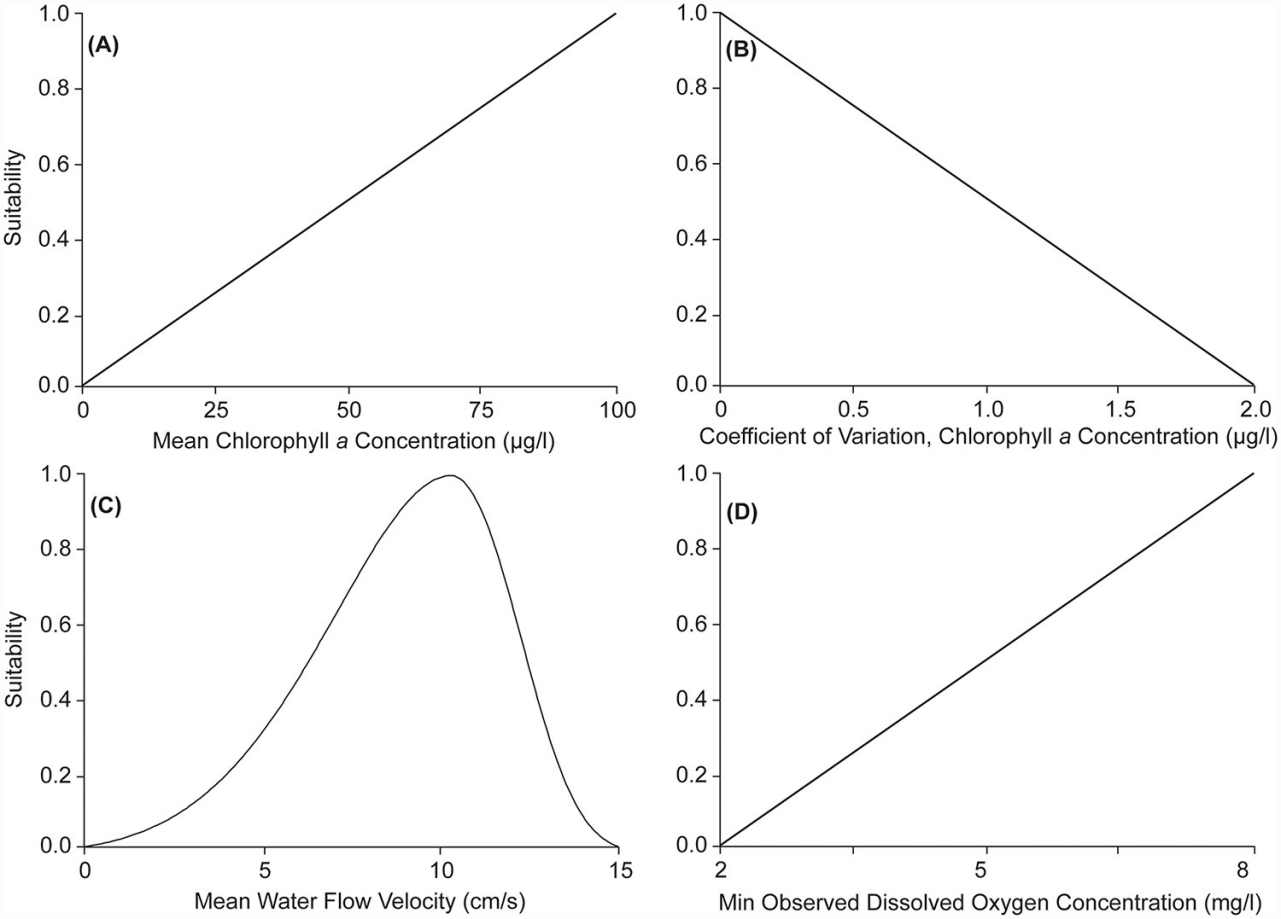
Relationship between actual values of: (A) mean chlorophyll a concentration, (B) coefficient of variation of chlorophyll a concentration, (C) mean water flow velocity (cm/s), and (D) minimum observed dissolved oxygen concentrations, and their associated suitability values. A description of the analysis methods used to develop these functions can be found in ‘*Methods*, *Water Filtration Ecosystem Services Layer Development*’.

We developed and applied suitability functions that increase linearly with increasing mean chlorophyll *a* concentration (Fig 4A), and that decrease linearly with increasing coefficient of variation of chlorophyll *a* concentration (Fig 4B). These functions consider areas with high monthly mean chlorophyll *a* concentrations and low coefficient of variation (i.e., most stable, high food availability) to be most suitable for reef restoration to provide maximum filtration benefits. While previous laboratory and modeling studies have identified non-linear relationships between chlorophyll *a* concentrations and oyster filtration at fine spatiotemporal scales (e.g., hourly and at the scale of 10’s of meters; [37–39]), the applicability of these relationships to the present study is uncertain given the coarse spatial (i.e., average chlorophyll *a* concentration in 1 km^2^ grid cells over an ~ 6,000 km^2^ area of Pamlico Sound) and temporal resolution (i.e., monthly mean chlorophyll *a* concentration averaged over a nine year period) of the available data. Moreover, the thresholds for initiation and cessation of filtration identified in laboratory settings for a similar oyster species (e.g., 25 μg l^-1^ and 500 μg l^-1^, respectively for *Crassostrea gigas* [37]) were only observed in c. 15% of our monthly mean chlorophyll *a* data, and the threshold of feeding cessation was never reached (Fig 3A). If future studies can generate chlorophyll *a* data at finer spatiotemporal scales than the present study, then it may be appropriate to apply a piecewise or other non-linear suitability function (sensu Cerco and Noel [39]). A more detailed review of the literature surrounding the functional relationships applied to the spatial datasets in this study—including a description of appropriate functions to apply for datasets of varying spatiotemporal scales—is provided in the “Discussion.”

**Water flow velocity** (cm s^-1^) data was derived from the Advanced Three-Dimensional Circulation Model (ADCIRC), a nonlinear, finite-element hydrodynamic model developed by Luettich et al. (1992) and validated in Pamlico Sound and adjacent estuarine and coastal waters of North Carolina by [40–42]. ADCIRC solves the shallow water form of the momentum equations over the entire APES domain represented by an unstructured grid developed by Reyns et al. [42] consisting of 22,425 nodes and 41,330 elements (resolution = 0.3–1 km) [43]. ADCIRC is forced with hourly wind velocities. To determine times of ‘average,’ ‘strongest,’ and ‘weakest’ winds corresponding with the range of wind conditions possible within the APES, we conducted residual sums of squares (RSS) analysis (sensu [44]) on wind speed and direction recorded at the Cape Hatteras Meteorological Station for September of 2012–2016 (i.e., 5 most recent years of available data provided by the Climate Office of North Carolina). During this period, ‘average,’ ‘strongest,’ and ‘weakest’ winds corresponded with September of 2014, 2015, and 2012, respectively. The model was subsequently forced with hourly wind velocities for these three monthly time periods. Bottom current velocities were output at hourly intervals for each of the 22,425 nodes. For each node, we subsequently calculated a: 1) mean water velocity, and 2) percent frequency of water velocities exceeding 15 cm s^-1^ (hereafter ‘exceedance frequency’). We evaluated the form of the relationship between mean water velocity and exceedance frequency using the loess function in R [36]. The relationship from the loess fit was nonlinear and sigmoid, which was confirmed after fitting a statistically significant four-parameter logistic function to the data within a global curve-fitting program (Fig 3B; [45]). As mean water velocity and exceedance frequency were significantly correlated, we developed a single spatial layer to represent water flow velocity suitability. Based on the identified inflection point of the sigmoid relationship between mean water velocity and exceedance frequency (i.e., 10.17 cm s^-1^), we generated and applied a left-skewed Weibull function to describe the relationship between mean water velocity and suitability for oyster water filtration. The form of this function yields increasing suitability to mean water velocities up to 10.17 cm s^-1^, and decreasing suitability beyond. At the inflection point of 10.17 cm s^-1^ at which suitability was decreased, exceedance frequency was 20–50% of the time (Fig 4C).

**DO concentration** (mg l^-1^) was derived from benthic DO concentration point measurements taken within the study area during the fall portion (i.e., corresponding with timing of peak hypoxia) of the North Carolina Division of Marine Fisheries Program 195, a fishery-independent trawl survey program (North Carolina Division of Marine Fisheries, personal communication). Data were collected at 522 unique stations between September of 1996 through 2014 (~52 randomly chosen stations sampled per year during September). As not all stations were revisited at the same interval, we considered the minimum observed DO concentration for each station in subsequent analyses. We utilized ordinary kriging within ArcMap [28] to generate an interpolated minimum DO concentration layer for the study area (i.e., a conservative estimate of DO). We applied a function that considered suitability to increase linearly with DO concentration, such that areas of highest observed DO were most suitable and areas of lowest DO were least suitable (Fig 4D).

### HSI integration

Similar to the development of the ‘Metapopulation Persistence HSI’ [18], we utilized expert stakeholder input (e.g., local academics [University of North Carolina at Chapel Hill Institute of Marine Sciences, North Carolina State University], non-governmental organizations [The Nature Conservancy], and state resource managers [North Carolina Division of Marine Fisheries]) to assign percentage weights to each of the individual layers considered within an updated HSI that simultaneously maximizes for both water filtration ecosystem services and metapopulation persistence (i.e., ‘Water Filtration & Metapopulation Persistence HSI;’ Table 1). Layer weights reflected expert stakeholders perceived relative importance of each layer for water filtration and metapopulation persistence (higher weight = more important). We generated weights for the ‘Water Filtration HSI’ by proportionally rescaling the assigned weights for the four oyster water filtration ecosystem service-associated layers (i.e., chlorophyll *a* mean, chlorophyll *a* variation, flow mean and variation, and dissolved oxygen) such that they summed to ~70% of the overall model for direct comparison with the ‘Metapopulation Persistence’ optimized HSI (Table 1), wherein four layers (salinity, larval export, larval import and dissolved oxygen; Table 1) accounted for 70% of that overall model. Other secondary considerations within the ‘Water Filtration HSI’ focused primarily on increasing oyster densities within a given reef location (i.e., larval export), minimizing potential predation and disease stressors (i.e., salinity), and certain logistical considerations (e.g., proximity to reef-building material stockpile sites).

### Model sensitivity

To determine and compare the sensitivity of the three HSI models to individual layers, we: 1) sequentially removed the four threshold layers with the highest weight, 2) re-weighted the remaining layers proportionally based on the weight of the removed layer, 3) re-ran the models, and 4) calculated the percent change in suitability of each cell (*S*_*j*_) with removal of each layer. This sensitivity analysis was conducted to quantify the relative importance of various highly layers within each model in part to inform efficient allocation of resources to gather accurate spatial data for certain factors that the model output is most sensitive to.

To determine the sensitivity of the three HSI models to our weightings, we ran the model with equal weightings for each of the threshold layers considered within each respective model (i.e., null model). As above, we similarly removed the four threshold layers with the highest weight from each null model and compared the impact of layer removal between final HSIs and the respective null models. This component of the sensitivity analysis was conducted to gauge the degree to which stakeholder-derived layer weightings influenced model output. It is important to note that a more positive percentage change (i.e., higher sensitivity) does not indicate an increase in habitat quality, but rather a greater change in habitat quality with removal of a variable. The change could increase or decrease habitat quality (i.e., suitability).

## Results

### Water Filtration Ecosystem Service Layers

#### Chlorophyll *a* concentration

**Fig 5:**
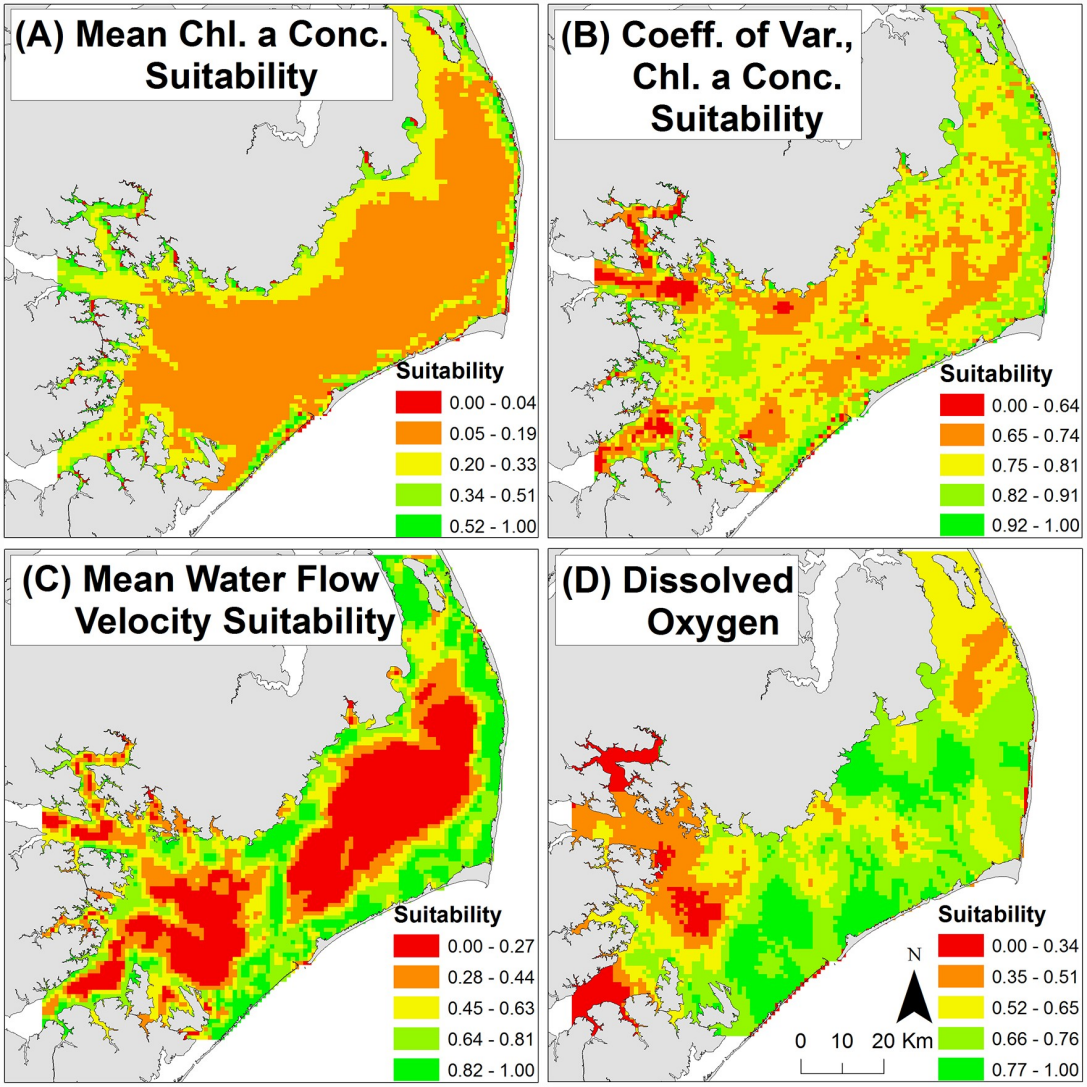
Suitability layer for: (A) mean chlorophyll a concentration, (B) coefficient of variation of chlorophyll a concentration, (C) mean water flow velocity, and (D) minimum observed dissolved oxygen concentration. Suitability for oyster restoration increases from low (red) to high (green) HSI.

Chlorophyll *a* concentrations varied widely spatially across Pamlico Sound and temporally across September 2003–2011 (i.e., S3 Fig depicts the wide range in September chlorophyll *a* concentrations across years). Mean September chlorophyll *a* concentrations across the nine-year time series and across the 5,987 grid cells contained within the model domain ranged from 9.12–98.98 μg chl *a* l^-1^, whereas coefficient of variation of September chlorophyll *a* concentrations ranged from 0.01–1.67. Mean chlorophyll *a* concentrations were generally highest and most variable near-shore within the bays, tributaries, and rivers flowing into Pamlico Sound (Fig 5A and 5B). Chlorophyll *a* concentrations were generally lowest and least variable within the central portion of Pamlico Sound (Fig 5A and 5B). Importantly, despite these general spatial patterns, mean chlorophyll *a* concentration and the coefficient of variation were uncorrelated (Fig 3A, *R*^*2*^ = 0.008).

#### Water flow velocity

Mean bottom current flow velocities were generally greatest in the shallow near-shore environment of Pamlico Sound, however near-shore locations generally exceeded 15 cm s^-1^ frequently. Deeper portions of the major bays, tributaries, and rivers flowing into Pamlico Sound, along with the central portion of the sound, generally had lower mean bottom current flow velocities. Suitability of mean water flow velocity was generally greater in shallow near-shore locations and lower in deeper waters (Fig 5C). Mean September bottom current flow velocities across the three-year time series and across the 22,425 nodes contained within the model domain ranged from 0.58–18.26 cm s^-1^. Mean September exceedance frequency (i.e., percent frequency flow velocity exceeds 15 cm s^-1^) ranged from 0–52%.

#### DO concentration

Minimum observed benthic DO concentrations ranged from 2–8 mg l^-1^ and were generally lowest within the major rivers flowing into Pamlico Sound and near their confluence in southwestern Pamlico Sound (Fig 5D). DO concentrations were generally highest and most suitable within the central portion of Pamlico Sound.

### Model simulations

#### Water filtration HIS

Suitability patterns of the ‘Water Filtration HSI’ (Fig 2) were driven largely by mean chlorophyll *a* concentration, DO concentration, water flow velocity, and coefficient of variation of chlorophyll *a* concentration—these four layers had a combined weighting of 73% (Table 1). Highly suitable restoration locations were primarily nearshore and located in the northwestern and western portions of Pamlico Sound, with some additional highly suitable areas in the southern and southwestern portions of the sound.

#### Water filtration & metapopulation persistence HIS

Suitability patterns of the ‘Metapopulation Persistence and Water Filtration HSI’ (Fig 2) were driven largely by mean chlorophyll *a* concentration, salinity, dissolved oxygen concentration, and larval export from oyster sanctuaries (i.e., settlement location of oyster larvae spawned from existing oyster sanctuaries). These four layers had a combined weighting of 55% (Table 1). Highly suitable restoration locations based on this HSI scenario balancing both metapopulation persistence- and water filtration-related criteria were located similarly to the ‘Metapopulation Persistence HSI’ in the southwestern and northwestern portions of Pamlico Sound (Fig 2). These suitable locations were generally more near-shore and included a greater portion of western Pamlico Sound than in the ‘Metapopulation Persistence HSI’.

#### Metapopulation persistence HIS

Suitability patterns of the original ‘Metapopulation Persistence HSI’ (Fig 2; Puckett et al. [18]) were driven largely by salinity, sanctuary larval export, sanctuary larval import (i.e., natal location of oyster larvae settling within existing oyster sanctuaries), and dissolved oxygen—these four layers had a combined weighting of 69% (Table 1). Highly suitable restoration locations based on the metapopulation persistence-related criteria considered within this model were located in the southwestern (mouth of Neuse and Pamlico Rivers and bays) and northwestern portions of Pamlico Sound (Fig 2). A detailed description of model output, suitability drivers, sensitivity analysis, and model validation results for this model is provided in Puckett et al. [18].

### Optimal restoration locations

**Fig 6:**
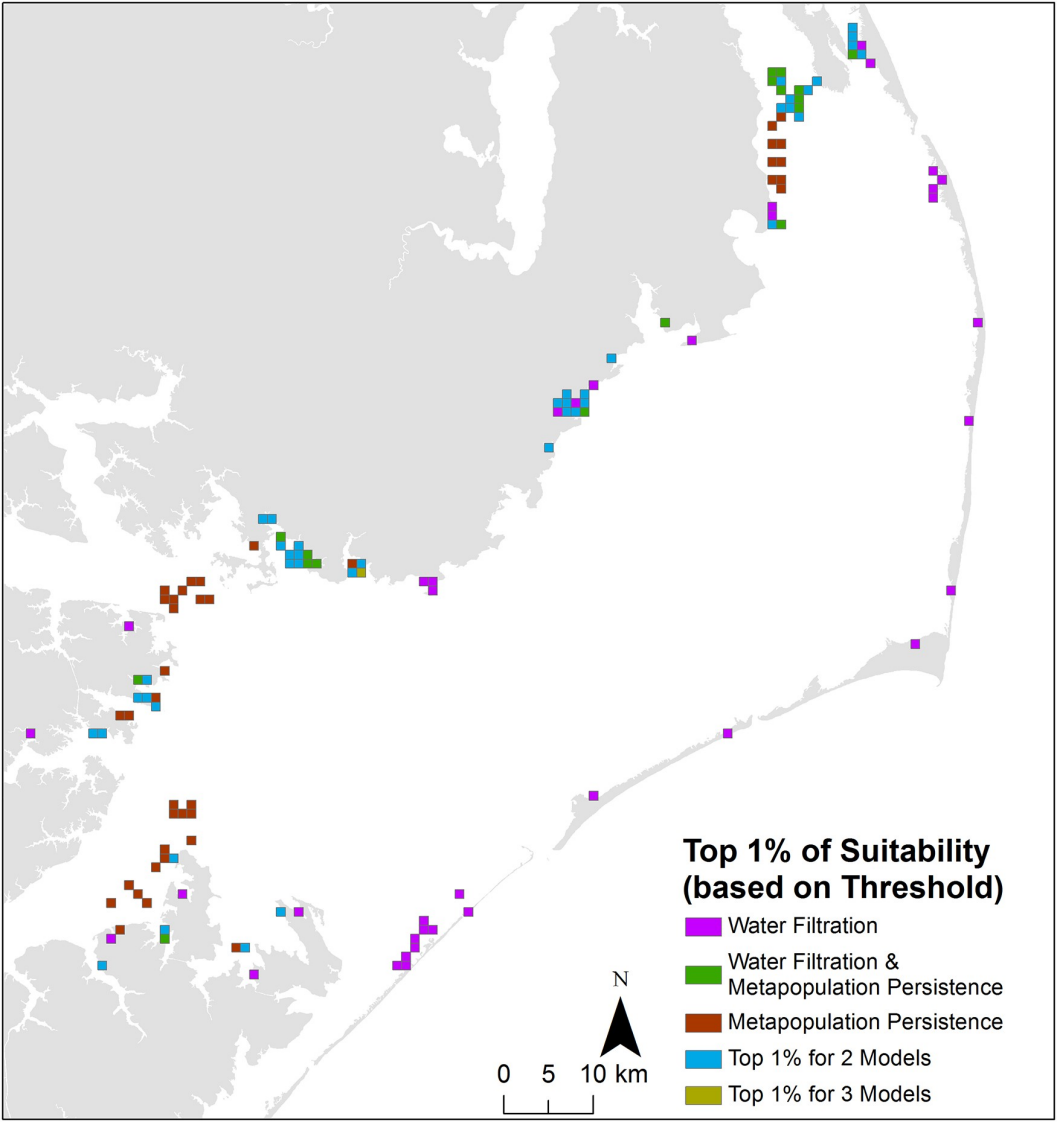
Optimal locations for restoration (i.e., top 1% highest HSI scores) identified in each HSI scenario as derived from the final HSI (i.e., aggregated threshold combined with aggregated exclusion layers). Locations that were identified within the top 1% for multiple HSI scenarios are indicated in blue (2 models) and gold (3 models). For example, ‘Top 1% for 2 Models’ may include areas identified within the top 1% for the ‘Water Filtration’ and the ‘Water Filtration & Metapopulation Persistence’ HSI scenarios, whereas ‘Top 1% for 3 Models’ includes areas identified within the top 1% for all three HSI scenarios.

Suitability scores associated with optimal restoration locations (defined as the top 1% of suitability scores) identified within the three HSI scenarios ranged from: 0.63–0.55 in the ‘Water Filtration HSI,’ 0.61–0.52 in the ‘Water Filtration & Metapopulation Persistence HSI,’ and 0.69–0.52 in the ‘Metapopulation Persistence HSI.’ Optimal locations within the ‘Water Filtration HSI’ were primarily located nearshore in the northwestern and western portions of Pamlico Sound, with two additional optimal locations near Ocracoke Inlet (Fig 6). Within the ‘Metapopulation Persistence HSI,’ optimal locations were primarily located within southwestern Pamlico Sound near the mouths of the Neuse and Pamlico Rivers. Optimal locations within the ‘Water Filtration & Metapopulation Persistence HSI’ overlapped entirely with optimal locations identified within the two other HSI scenarios (i.e., contained within the ‘Top 1% for 2 & 3 Models’ categories in Fig 6). Optimal locations identified within two or three HSI scenarios (i.e., ‘win-win’ restoration locations) were located primarily within northwestern and western Pamlico Sound (Fig 6).

### Model sensitivity

**Fig 7:**
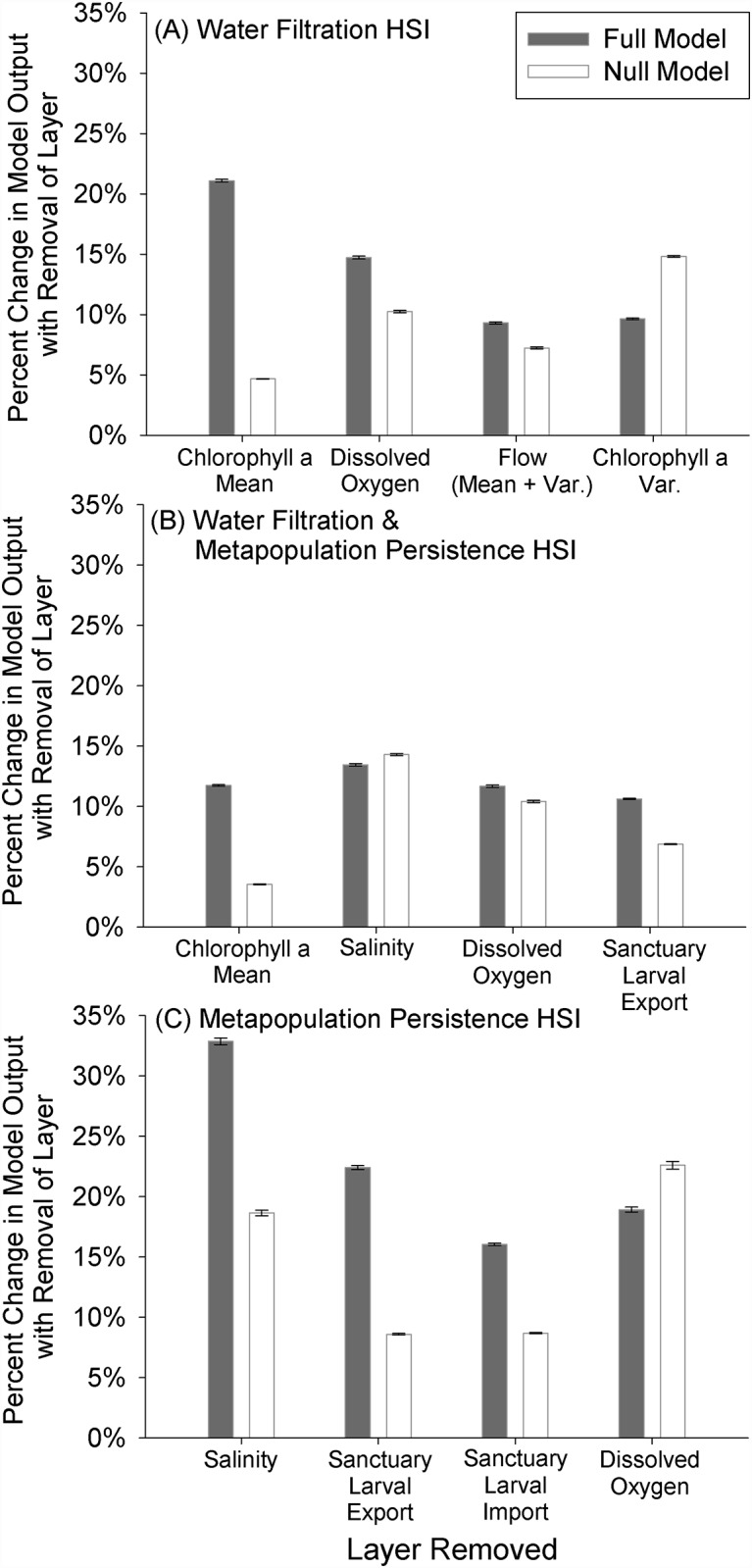
Results of model sensitivity analysis conducted for each of the three HSI scenarios where we: 1) removed the four threshold layers with the highest weight individually, 2) re-weighted the remaining layers proportionally based on the weight of the removed layer, 3) re-ran the model, and 4) calculated on a cell-by-cell basis the percent change in model output with removal of each layer. Error bars represent standard error of the mean (n = 5,987) of the percent change in model output with removal of a layer. Weights associated with the threshold layers in each HSI scenario can be found in Table 1.

The ‘Water Filtration HSI’ was generally most sensitive to layers in order of their weightings, except for the coefficient of variation of chlorophyll *a* layer (9.66%), which the model was more sensitive to removal of than the water flow velocity layer (9.32%; Fig 7A). The percent change in HSI averaged among all grid cells was 21.10% +/- 0.11% SE with the removal of the mean chlorophyll *a* layer (weighted at 28%) and 14.73% +/- 0.12% SE with removal of the dissolved oxygen layer (weighted at 19%). The null model, with equal layer weightings, was most sensitive to removal of the chlorophyll *a* variation layer, followed by dissolved oxygen, water flow velocity, and chlorophyll *a* mean.

In general, the ‘Water Filtration & Metapopulation Persistence HSI’ was equally sensitive to each of the top four highest weighted layers (Fig 7B), with a marginally greater sensitivity to the removal of the salinity layer. The percent change in HSI with the removal of the mean chlorophyll *a* layer (weighted at 17%) and averaged among all grid cells was 11.73 +/- 0.07 SE%, and 13.45 +/- 0.10 SE% with removal of the salinity layer (weighted at 15%). The null model with equal layer weightings was most sensitive to removal of the salinity layer, followed by dissolved oxygen, sanctuary larval export, and chlorophyll *a* mean.

The ‘Metapopulation Persistence HSI’ was most sensitive to layers in order of their weightings, except for the dissolved oxygen layer, which the model was more sensitive to removal than the sanctuary larval import layer (Fig 7C). The percent change in the HSI after the removal of the salinity layer (weighted at 23%) and averaged among all grid cells was 32.9 +/- 0.2 SE%, and 22.4 +/- 0.2 SE% with removal of the oyster larval export layer (weighted at 20%). The null model with equal layer weightings was most sensitive to removal of the dissolved oxygen layer (i.e., layer with the greatest degree of spatial variability), followed by salinity, sanctuary larval import, and sanctuary larval export.

## Discussion

Habitat suitability indices (HSI) are valuable, quantitative tools to guide spatial planning of habitat restoration efforts in locations with the greatest potential for success [5, 6, 17]. These models, however, have generally not incorporated factors of direct relevance to siting restoration in locations that would maximize ecosystem service provision. Furthermore, given the financial costs and varying goals attributed to specific habitat restoration projects (e.g., restoring oyster reefs to support oyster fishery harvest, provide shoreline stabilization benefits, or to provide essential fish habitat / recreational fishing opportunities), multiple HSI models optimized for multiple restoration goals within a given waterbody are warranted to identify locations that are ‘win-win’ and locations where ‘tradeoffs’ among management goals must be considered. Novel variables associated with oyster water filtration ecosystem services were integrated within an existing, ‘Metapopulation Persistence’ optimized GIS-based HSI model containing biophysical (e.g., salinity, oyster larval connectivity) and logistical (e.g., distance to nearest restoration material stockpile site) variables to identify suitable locations for oyster restoration that maximize long-term persistence of restored oyster populations and water filtration ecosystem service provision. Furthermore, the ‘Water Filtration’ optimized HSI was compared to the HSI optimized for ‘Metapopulation Persistence,’ as well as a hybrid model that optimized for both water filtration and metapopulation persistence. We compared both suitability patterns and optimal locations (i.e., locations corresponding to the top 1% of suitability score) identified within and between three HSI scenarios optimized for varying restoration goals. The conceptual framework utilized in this study, wherein restoration goal-specific HSIs were developed and “win-win” (i.e., optimal restoration locations identified in multiple goal-specific HSIs) versus “tradeoff” (i.e., optimal restoration locations identified in a single goal specific HSI) restoration locations were identified, can inform development of similar restoration goal-specific HSI models in other systems.

Traditional approaches to site selection for oyster restoration have been based, in part, on locations where oysters were historically abundant (e.g., maps of historic distribution and abundance [18]). However, substantial changes to estuarine ecosystems over time may preclude the utility of historical records for present-day site selection rendering HSI models that incorporate modern factors relevant to restoration site selection increasingly valuable. Theuerkauf and Lipcius [17] conducted a review of HSI models developed for aquaculture, fishery production, and restoration of oyster species and found that most oyster HSI models focused on incorporation of biophysical factors essential to reef persistence, but lacked logistical or ecosystem services factors of relevance to identification of compatible locations for oyster reef restoration. Oyster HSI models are generally developed with a focus on physical factors such as salinity, substrate type, and water depth (e.g., [17, 46]). We included these core factors within our model with substrate type and water depth being binary exclusion layers, and salinity serving as a highly weighted threshold layer in the model due to its importance in several oyster biological processes [9]. To address the increasing desire to restore oyster reefs to recover lost ecosystem services, we also included several novel layers relevant to oyster water filtration service provision including chlorophyll *a* concentrations, water flow velocities, and dissolved oxygen concentrations. The advantages of this approach to identifying optimal restoration locations are that we incorporate biophysical factors that are important for reef persistence, which is a common goal in similar oyster-based HSI models, while also integrating several factors relevant to an important ecosystem service. This enabled us to not only guide ecosystem-service based restoration, but also to evaluate optimal locations for restoration across three different restoration goal scenarios, which is a novel advance in the use of HSIs for restoration. Further and more broadly, incorporation of logistical and ecosystem service factors should be an important goal within HSI models for habitat restoration given the importance of these considerations to restoration practitioners as identified within our stakeholder engagement process used to guide development of our HSI models.

There are limitations to the novel data layers included within the present study due to uncertainties around the functional relationships between biotic and abiotic parameters, oyster performance (e.g., feeding rate, physiological stress, etc.), and the relative appropriateness of applying differing relationships depending on the spatiotemporal scale of available data. For example, chlorophyll *a* concentrations, water flow velocities, and dissolved oxygen concentrations were selected to generate oyster water filtration ecosystem services layers because: 1) these factors have been identified within the literature as important to oyster filtration, and 2) spatially-explicit datasets were available for our study system. Chlorophyll *a* serves as a reliable surrogate for phytoplankton biomass [47], and previous field and modeling research identified a significant, positive relationship between chlorophyll *a* concentrations and oyster growth rates [29, 46]. Laboratory experiments that examined bivalve feeding under varying food particle concentrations identified both minimum and maximum particle concentrations that generate an “on-off switch” for filtration [37]. Below a minimum particle concentration threshold (e.g., 25 μg l^-1^ for *Crassostrea gigas*), active filtration is energetically unfavorable as caloric expenditure exceeds caloric uptake [38]. Above a critical particle concentration threshold (e.g., 500 μg l^-1^ for *Crassostrea gigas*), filtration is also energetically unfavorable as excess energy is expended to clear clogged mucus from the gills. Previous fine-scale oyster filtration modeling efforts have incorporated this minimum and maximum particle concentration “on-off” switch. For example, Cerco and Noel [39] utilized a piecewise function to parameterize oyster filtration rates under varied suspended solid concentrations (i.e., reduced filtration rates at low and high total suspended solids concentrations, highest filtration rates at a moderate total suspended solids concentration). It is probable that, under field environmental conditions wherein particle concentrations fluctuate rapidly as a function of water flow speed and direction, individual bivalves mediate between periods of active filtration and inactivity depending upon ambient particle concentrations—likely on the scales of minutes to hours.

Within the present study, given the coarse spatiotemporal resolution of available chlorophyll *a* data and uncertain applicability of previously observed fine-scale relationships, we applied linear suitability functions that consider areas with high monthly mean chlorophyll *a* concentrations and low coefficient of variation to be most suitable for reef restoration to provide maximum filtration benefits (i.e., most stable, high food availability). However, if a piecewise suitability function were applied to simulate an “on-off” switch for filtration, chlorophyll *a* concentrations above the minimum concentration threshold (e.g., 25 μg l^-1^ for *Crassostrea gigas* [38]) and below the maximum concentration threshold (e.g., 500 μg l^-1^ for *Crassostrea gigas*) could be considered as highly suitable, and those outside of the range as unsuitable (i.e., binary). Application of such an “on-off” suitability function would be of limited use at a maximum chlorophyll a concentration of 500 μg l^-1^ (i.e., the “off” switch) because we observed no mean monthly chlorophyll *a* values above 100 μg l^-1^. At the minimum chlorophyll *a* concentration of 25 μg l^-1^, the impact on suitability would be limited because values less than this are scored as largely unsuitable (i.e., scores close to 0) in the current linear model we applied. Application of an “on-off” suitability function would also increase the homogeneity of the chlorophyll *a* suitability layer and decrease the layer’s utility in assisting with the site selection process because c. 85% of the grid cells in our model domain were between 25 and 500 μg l^-1^ and, therefore, would be scored as highly suitable (i.e., assigned a score of ‘1’). Furthermore, given the importance of the minimum and maximum chlorophyll *a* concentration thresholds in determining suitable versus unsuitable locations, application of a piecewise function of this kind would need to be precisely parameterized according to the appropriate concentration thresholds for a particular species and waterbody. Field-based studies are needed to examine if this “on-off feeding switch” or other non-linear feeding relationship with chlorophyll *a* concentrations occur within individual oysters on reefs and the spatiotemporal scales at which it is relevant under realistic field conditions. These studies should further seek to evaluate the role of oyster filtration rates and capacity in areas along a gradient of mean phytoplankton biomass (i.e., areas of low to high mean chlorophyll *a* concentration).

Water flow velocities, combined with seston composition and concentration, can be significant drivers of overall oyster filtration capacity [48]. For example, increasing water flow velocities yields enhanced food delivery to individual oysters on reefs [11], yet water flow velocities above 15 cm s^-1^ can inhibit individual feeding and growth [48, 49]. The exact causal mechanisms underlying this threshold remains unknown and should be the subject of future research; however, it is probable that high flow velocities (i.e., > 15 cm s^-1^) can result in turbulent resuspension of reef sediments that may trigger cessation of filtration (i.e., exceedance of the maximum particle concentrations above which filtration is energetically favorable [11, 37]).

Hypoxic or anoxic conditions are increasingly common within urbanized estuaries because of eutrophication [50]. Low dissolved oxygen conditions within bottom waters can yield lethal impacts to benthic organisms, such as oysters [51]. Thus, incorporation of spatial information on dissolved oxygen concentrations is an essential consideration when developing spatial guidance tools for restoration of benthic organisms that incorporate indicator variables of phytoplankton biomass (e.g., chlorophyll *a* concentrations). We conservatively estimated minimum benthic dissolved oxygen concentrations throughout Pamlico Sound and applied a linear suitability function that characterized areas of estimated lowest minimum benthic dissolved oxygen concentration as least suitable for oyster restoration and areas of highest as most suitable. Visual comparison of the major patterns of lowest benthic dissolved oxygen within the major rivers flowing into Pamlico Sound and near their confluence in southwestern Pamlico Sound (Fig 5D) aligns with previous research that has observed similar patterns of low dissolved oxygen within the depths of these rivers, particularly the Neuse, Pamlico and Pungo Rivers, which exhibit the highest frequency of fish kills in North Carolina, and that have been attributed to widespread benthic hypoxia and anoxia [52, 53]. We further provide a visualization of kriging variance (S4 Fig), which provides an assessment of where predicted estimates of benthic dissolved oxygen concentration have the greatest uncertainty. The highest observed kriging variance occurred within the upper portion of minor tributaries to Pamlico Sound and along the shallow portions of Pamlico Sound along the Outer Banks barrier islands, due in large part to a lack of sampling stations in these areas. Where data are available at an improved spatiotemporal scale (e.g., hourly observations at sampling stations spaced ~1 km apart across a waterbody), a binary suitability function (similar to those applied to ‘exclusion’ layers in this study) could be appropriate to consider areas of benthic hypoxia (e.g., < 4 mg l^-1^) as unsuitable (i.e., assigned a score of ‘0’) and other areas as suitable (i.e., assigned a score of ‘1’). In our study, application of this binary function yielded a resultant suitability layer that inappropriately considered areas of known episodic hypoxia as suitable (S5 Fig). For instance, in the lower Neuse River and in northeastern Pamlico Sound (i.e., near Manteo, North Carolina) we deployed YSI 6600 water quality sondes during the summer of 2012 and observed extended periods (1–2 weeks) of hypoxic conditions in the areas identified as suitable in the binary version of the dissolved oxygen suitability layer (D. Eggleston unpublished data). Application of the previously described linear suitability function more adequately captures areas of known episodic hypoxia.

Water temperature, turbidity, freshet frequency, and salinity variation are additional environmental parameters that have been included in previous HSI models [46, 54–56]. These factors are relevant for consideration in future ‘Water Filtration’ optimized HSI models, but data of sufficient spatial and temporal resolution were unavailable for the present study. For example, oyster filtration rates are constant between 16–28 °C, but increase rapidly above 28 °C [57]. In all cases, we recommend careful consideration of the spatiotemporal scale of available data and the functional form of suitability functions.

We defined optimal restoration locations as those locations corresponding to the top 1% of suitability scores for three reasons: (1) to narrow the number of highest priority potential restoration locations for restoration practitioners, (2) to convey the observed spatial variation in identified optimal locations for restoration across the three HSI scenarios, and (3) for comparison with the results of Puckett et al. [18]. Reduction of the model results to a narrow subset of locations (i.e., top 1% of suitability scores) is an important outcome of this study as restoration practitioners often have limited time and funding to conduct field verification and ground-truthing within predicted highly suitable locations, and communication of this narrow subset of locations allows for more efficient prioritization of resources. Overall suitability patterns varied spatially between the three HSI scenarios. Within the ‘Water Filtration HSI’ (Fig 2A and 2E), highly suitable restoration locations were concentrated primarily nearshore within the northwestern and western portions of Pamlico Sound, driven largely by the highly suitable chlorophyll *a*, water flow velocities, and dissolved oxygen conditions that co-occur in those areas. Within the ‘Metapopulation Persistence HSI’ (Fig 2C and 2G), highly suitable restoration locations were located in the southwestern (mouth of Neuse and Pamlico Rivers and bays) and northwestern portions of Pamlico Sound, driven largely by the highly suitable salinity and larval connectivity with oyster sanctuaries in those areas. The ‘Water Filtration & Metapopulation Persistence’ HSI (Fig 2B and 2F), given its incorporation of factors from both the ‘Water Filtration’ and the ‘Metapopulation Persistence’ HSI models, largely reflected an average of the suitability patterns observed in those two HSI scenarios (i.e., high suitability nearshore within the northwestern, western, and southwestern portions of Pamlico Sound). For additional comparative purposes, we provide a map representing the locations corresponding to the top 5% of suitability scores which reflect the same general patterns as described above for the optimal locations identified using the top 1% of suitability scores for each HSI scenario (S6 Fig). Comparison of predicted suitable habitat from each of the three HSI scenarios in relation to historic oyster reef distribution based on Lt. Francis E. Winslow’s 1887–1887 survey of subtidal oyster reefs in Pamlico Sound indicates a degree of congruence between historic distribution and highly suitable locations as identified within the ‘Water Filtration HSI’ (S7 Fig) [18]. Mismatches between the distribution of historical subtidal oyster reefs and predictions of suitable habitat for oyster sanctuary restoration as identified by each HSI scenario could be due to: (1) changes in salinity patterns from opening and closing of inlets, (2) increases in hypoxia and anoxia affecting the tributaries and sub-estuaries of Pamlico Sound, (3) discrepancies in the scope of the HSI models (i.e, siting oyster sanctuaries to optimize various restoration goals) and the historic data being used for comparison (natural subtidal reefs), among other potential factors [18].

Model sensitivity analyses and quantitative model validation are important steps in the HSI model development process [17, 18]. We quantitatively evaluated the sensitivity of each HSI model to individual layers and their assigned weightings (Table 1, Fig 7). The order of sensitivity of each full HSI model to layer removal generally followed the corresponding order of the assigned layer weights (i.e., a greater percent change in model output with removal of higher weighted layers), with a few notable exceptions. For example, in the ‘Water Filtration’ HSI (Fig 7A), a greater percent change in model output was observed with removal of the chlorophyll *a* variation layer relative to the higher weighted flow velocity layer. This enhanced sensitivity to layer removal is likely due to the more spatially dynamic nature of certain layers within these models (i.e., high degree of spatial heterogeneity with considerable small- and large-scale variability) relative to the other more highly weighted layers. The impact of the greater degree of spatial heterogeneity associated with certain layers was further emphasized in the sensitivity analysis of the null models (i.e., where all layers are weighted equally). For example, within the ‘Water Filtration’ HSI null model, removal of the highly spatially heterogeneous chlorophyll *a* variation layer (Fig 5B) resulted in the greatest percent change in model output with layer removal relative to other layers considered within the model. These results highlight the importance of acquiring spatial data layers for parameters that are spatially dynamic (e.g., dissolved oxygen, chlorophyll *a*) when developing similar HSI models for other systems. In all three HSI scenarios, the null, equally-weighted model was less selective than the full model (S7 Fig). The null models were characterized by a unimodal distribution of HSI values and limited clustering of optimal restoration locations, whereas the full models had bimodal distributions of HSI values and greater clustering of optimal locations.

Rigorous validation of the ecosystem services-optimized HSI models presented here would require construction of reefs along a gradient of suitability (e.g., constructing reefs in areas of high to low suitability within each HSI scenario) and assessment of response variables of interest (e.g., oyster density, growth and survival rates, chlorophyll *a* reduction). Thus, quantitative validation of the ecosystem services-optimized HSI models presented here is beyond the scope of this study (but see Puckett et al. for model validation results for the ‘Metapopulation Persistence’ HSI wherein normalized oyster densities on existing oyster sanctuaries were found to be a positive exponential function of HSI score [18]). However, the functional relationships between oyster water filtration and chlorophyll *a* concentrations, water flow velocities, and dissolved oxygen developed in this study can guide field- and lab-testing of hypotheses related to optimal conditions for oyster reef restoration to maximize water quality enhancement benefits. For example, as described above, field-based studies are needed to examine if a threshold particle concentration “on-off switch” occurs within individual oysters on reefs and, if so, its impact on oyster filtration rates and capacity in areas along a gradient of mean phytoplankton biomass. Information from these proposed studies could be used to update the functional relationships between oyster water filtration and chlorophyll *a* concentrations, water flow velocities, and dissolved oxygen (Fig 4) developed and utilized in this study or other similar models in the future. Thorough literature reviews were an essential component in the development of the suitability functions used within this ecosystem services HSI. Future studies that incorporate ecosystem services considerations within HSI models should utilize relevant results from field- and lab-based studies to develop meaningful suitability functions that relate biophysical parameters with the capacity of a species or habitat to provide ecosystem services.

The development of goal-specific HSI models (e.g., maximizing long-term metapopulation persistence, maximizing ecosystem service provision) can (1) provide researchers with information on knowledge gaps with respect to the underlying functional relationship between biotic or abiotic factors, and target restoration response variables for restored habitats, and (2) provide restoration practitioners with valuable spatial information to guide where habitat restoration efforts might be most successful for one or more restoration goals. Implementation of the results of this study requires restoration practitioners to clearly define restoration goals (e.g., restoration to provide metapopulation enhancement value) and other constraining criteria (e.g., focusing restoration only within a specific portion of the system) a priori to determine which optimal restoration locations as identified from an HSI scenario are compatible. Secondary considerations (such as a desire to spread restoration efforts throughout a waterbody) should also guide the determination of which optimal restoration locations are compatible and to assist with decision making when tradeoffs are being considered (e.g., selection of an optimal location for one restoration goal over another). It is important to note that the results of HSI models are valuable for identifying specific locations with the greatest likelihood of restoration success and maximization of restoration goals, however, rigorous field surveys and validation are still necessary to ensure appropriateness of a specific location for restoration.

The approach applied in this study, and originally developed by Puckett et al. [18], provides a useful case study wherein stakeholder input was used directly to shape model development and parameterization that, in-turn, enhanced stakeholder ‘buy-in” to the modeling approach and adoption of the model output for restoration planning. By directly engaging stakeholders and restoration practitioners in the development process from the outset, these models have been successfully implemented and utilized by restoration practitioners in North Carolina to inform restoration siting [58]. For example, the ‘Metapopulation Persistence’ HSI was utilized by the North Carolina Division Marine Fisheries to inform the site selection process for the Swan Island oyster sanctuary constructed in Pamlico Sound in 2017. Further, the conceptual framework and methods used in this study, wherein HSIs were developed to meet specific restoration goals (e.g., maximizing long-term metapopulation persistence, maximizing ecosystem service provision), can broadly inform development of similar restoration goal-specific HSI models in other systems.

## Supporting information

S1 Table List of 17 GIS layers used originally by Puckett et al., 2018 and in the present study to determine suitability of sites in Pamlico Sound, North Carolina USA for placement of oyster reefs to maximize the probability of population persistence (i.e., ‘Population Persistence HSI’). Threshold layers were assigned thresholds (e.g., optimal [score = 1], suitable [0.5], and unsuitable [0]) and subsequently weighted based on the layer’s relative importance. A stakeholder panel was used to assign thresholds and weights. Exclusion layers were binary (suitable [score = 1] or unsuitable [0]). A detailed description of the methods used to develop these layers is provided in Puckett et al., 2018. Layer abbreviations are as follows: Submerged Aquatic Vegetation (SAV). Source abbreviations are as follows: National Oceanic and Atmospheric Administration (NOAA), North Carolina Division of Marine Fisheries (DMF), North Carolina Department of Environmental Quality (DEQ), United States Geological Survey (USGS), North Carolina Wildlife Resource Commission (WRC).
(DOCX)

S2 Table Mean (± standard error of the mean) monthly-averaged chlorophyll *a* concentration for Pamlico Sound across the nine-year MERIS satellite imagery data series (2003–2011) used to determine the timing of average peak phytoplankton biomass within the system. Chlorophyll *a* concentrations were averaged across all pixels for a given sampling period to obtain a single value for a monthly mean chlorophyll *a* concentration and subsequently averaged across years within a given month (e.g., averaged across January 2003 through 2011). September, which corresponds with the fall phytoplankton bloom in Pamlico Sound, was determined to be the month of maximum average chlorophyll *a* concentration for the period of 2003 through 2011.
(DOCX)

S1 Fig Threshold layers used to compute habitat suitability for oyster restoration within the three HSI scenarios. A) Salinity. B) Sanctuary larval export. C) Sanctuary larval import. D) Cultch reef larval import. E) Natural reef larval import. F) Cultch reef larval export. G) Natural reef larval export. H) Material stockpile sites. I) Boat ramps. Suitability increases from low (red) to high (green) for each layer.
(TIF)

S2 Fig Exclusion layers used to compute habitat suitability for oyster restoration within the three HSI scenarios. A) Bathymetry. B) Bottom Type. C) SAV. D) Shellfish leases. E) Nursery areas. F) Military zones. G) Navigational channels. Suitability increases from unsuitable (red) to optimal (green) for each layer.
(TIF)

S3 Fig Monthly-averaged (i.e., averaging across all days within the month) chlorophyll *a* concentrations (microgram l^-1^) in Pamlico Sound corresponding with the (A) lowest overall, (B) closest to average, and (C) highest overall observed chlorophyll a concentrations as compared to all Septembers between 2003–2011. (TIF)

S4 Fig Kriging variance for interpolated minimum benthic dissolved oxygen concentrations in Pamlico Sound. Fall sound-wide minimum benthic dissolved oxygen concentrations from 1996–2014 were interpolated using ordinary kriging to estimate minimum benthic dissolved oxygen throughout the system.
(TIF)

S5 Fig Dissolved oxygen suitability as defined using a binary suitability function that considers areas of benthic hypoxia (e.g., < 4 mg l^-1^) as unsuitable (i.e., assigned a score of ‘0’) and other areas as suitable (i.e., assigned a score of ‘1’). Note that this layer was not utilized within the analysis as it inappropriately considers areas of known episodic hypoxia (e.g., within the Neuse and Pamlico Rivers) as suitable.
(TIF)

S6 Fig Optimal locations for restoration (i.e., top 5% highest HSI scores) identified in each HSI scenario as derived from the final HSI (i.e., aggregated threshold combined with aggregated exclusion layers). Locations that were identified within the top 5% for multiple HSI scenarios are indicated in blue (2 models) and gold (3 models). For example, ‘Top 5% for 2 Models’ may include areas identified within the top 5% for the ‘Water Filtration’ and the ‘Water Filtration & Metapopulation Persistence’ HSI scenarios, whereas ‘Top 5% for 3 Models’ includes areas identified within the top 5% for all three HSI scenarios.
(TIF)

S7 Fig Comparison of the null, equally-weighted model results for the three HSI scenarios, (A) Water Filtration, (B) Water Filtration and Metapopulation Persistence, and (C) Metapopulation Persistence with the full, stakeholder-weighted model results for the three HSI scenarios, (D) Water Filtration, (E) Water Filtration and Metapopulation Persistence, and (F) Metapopulation Persistence. The location of historic oyster reefs are depicted by black crosshatch.
(TIF)
