# Supplementary material for: Integrating ecosystem services considerations within a GIS-based habitat suitability index for oyster restoration
Source: PLoS One. 2019 Jan 25;14(1):e0210936. doi: 10.1371/journal.pone.0210936 (PMC6347164; doi:10.1371/journal.pone.0210936)
Supplement: S1 Table — Threshold layers were assigned thresholds (e.g., optimal [score = 1], suitable [0.5], and unsuitable [0]) and subsequently weighted based on the layer’s relative importance. A stakeholder panel was used to assign thresholds and weights. Exclusion layers were binary (suitable [score = 1] or unsuitable [0]). A detailed description of the methods used to develop these layers is provided in Puckett et al., 2018. Layer abbreviations are as follows: Submerged Aquatic Vegetation (SAV). Source abbreviations are as follows: National Oceanic and Atmospheric Administration (NOAA), North Carolina Division of Marine Fisheries (DMF), North Carolina Department of Environmental Quality (DEQ), United States Geological Survey (USGS), North Carolina Wildlife Resource Commission (WRC). (DOCX) [file pone.0210936.s001.docx]

| **Layer** | **Description** | **Type** | **Threshold and Exclusion Values** | **Rationale** | **Source** |
| --- | --- | --- | --- | --- | --- |
| **Salinity (psu)** | Summer sound-wide salinity during average freshwater input from 1987-2008. | Threshold | Optimal (1): 10-15  Suitable (0.5): 6-10; 15-25  Unsuitable (0): <6; >25    Thresholds based on values reported in Kennedy (1996). | Important to oyster biological processes (e.g., growth and survival). | NC DMF Trawl Survey Program; Durham 2009 |
| **Sanctuary Larval Export** | Settlement location of oyster larvae spawned from existing sanctuaries. | Threshold | Continuous (0-1)    Log-transformed abundance of larval settlers in a cell, standardized on a 0 (lowest abundance) to 1 (highest abundance) scale. | Goal for existing sanctuaries to export larvae to future sanctuaries to create sanctuary network. | Puckett et al. 2016; Puckett et al. 2018 |
| **Sanctuary Larval Import** | Natal location of oyster larvae settling within existing sanctuaries. | Threshold | Continuous (0-1)    Log-transformed abundance of larvae spawned in a cell, standardized on a 0 (lowest abundance) to 1 (highest abundance) scale. | Goal for existing sanctuaries to import larvae from future sanctuaries to create sanctuary network. | NC DMF Oyster Sanctuary Program; This Study |
| **Dissolved Oxygen** | Fall sound-wide minimum dissolved oxygen concentrations from 1996-2014. | Threshold | Continuous (0-1)    Minimum dissolved oxygen values linearly transformed on a 0 (lowest) to 1 (highest) scale. | Important to oyster biological processes (e.g., survival). | NC DMF Trawl Survey Program; This Study |
| **Cultch-planting Site Larval Import** | Natal location of oyster larvae settling within cultch-planting sites—locations where oyster shell is deployed to replace shell removed through commercial oyster harvest. Cultch-planting sites established from 2010-2014 were used. | Threshold | Continuous (0-1)    Log-transformed abundance of larvae spawned in a cell, standardized on a 0 (lowest abundance) to 1 (highest abundance) scale. | Goal for existing sanctuaries to export larvae to commercially harvested cultch-planting sites. Promotes larval exchange among strategies within the oyster restoration portfolio. | NC DMF Habitat Enhancement Program; This Study |
| **Natural Reef Larval Import** | Natal location of oyster larvae settling within natural subtidal reefs. | Threshold | Continuous (0-1)    Log-transformed abundance of larvae spawned in a cell, standardized on a 0 (lowest abundance) to 1 (highest abundance) scale. | Goal for existing sanctuaries to export larvae to commercially harvested natural reefs. | NC DMF Estuarine Benthic Habitat Mapping Program; This Study |
| **Cultch-planting Site Larval Export** | Settlement location of oyster larvae spawned from cultch-planting sites. Cultch-planting sites established from 2010-2014 were used. | Threshold | Continuous (0-1)    Log-transformed abundance of larval settlers standardized on a 0 (lowest abundance) to 1 (highest abundance) scale. | Enables selection of sites that promotes larval exchange among strategies within the oyster restoration portfolio. | NC DMF Habitat Enhancement Program; This Study |
| **Natural Reef Larval Export** | Settlement location of oyster larvae spawned from natural subtidal oyster reefs. | Threshold | Continuous (0-1)    Log-transformed abundance of larval settlers standardized on a 0 (lowest abundance) to 1 (highest abundance) scale. | Enables selection of sites that promotes larval exchange among natural and restored oyster reefs. | NC DMF Estuarine Benthic Habitat Mapping Program; This Study |
| **Material Stockpile Sites (km)** | Location of material stockpile sites accessed by vessels for loading material (e.g., reef balls) used for restoration. | Threshold | Optimal (1): <5 km  Suitable (0.5): 5-10 km  Unsuitable (0): >10 km    Thresholds based on vessel range (trips per day), fuel costs, and vessel load capabilities. | Enables selection of sites based on economic and logistical constraints associated with transporting large amounts of hard substrate needed for restoration. | NC DMF Oyster Sanctuary Program |
| **Boat Ramps (nm)** | Location of boat ramps where recreational fishermen can launch boats. | Threshold | Optimal (1): <10 km  Unsuitable (0): >10 km    Thresholds based on average travel distance reported for inshore recreational vessels by Ramos et al. (2006). | Enables selection of sites based on accessibility to recreational (fin)fishing. | NC WRC |
| **Bathymetry** | Depth (m) in Pamlico Sound. | Exclusion | Excluded if depth of entire 1km^2^ cell was < 2m, which is required for navigational clearance. | - | NOAA Estuarine Bathymetry |
| **Bottom Type** | Bottom sediment composition in Pamlico Sound. Bottom types included: sand, muddy sand, sandy mud, mud, and soft mud. | Exclusion | Excluded cells with soft sediment composition (e.g., soft mud and mud), which is necessary to prevent subsidence of heavy material used for reef restoration. | - | NC DMF Estuarine Benthic Habitat Mapping and Trawl Survey Programs |
| **SAV** | Location of SAV in Pamlico Sound. | Exclusion | Excluded if a cell contained SAV because reef restoration is not permitted in the presence of SAV. | - | NC DMF Estuarine Benthic Habitat Mapping Program |
| **Shellfish Leases** | Location of private shellfish leases in Pamlico Sound. | Exclusion | Excluded if a cell contained leased area to prevent user conflicts. | - | NC DMF Estuarine Benthic Habitat Mapping Program |
| **Nursery Areas** | Location of primary and special secondary nursery areas in Pamlico Sound, which serve as critical habitat for juvenile fishes. | Exclusion | Excluded if a cell contained primary or special secondary nursery areas because reef restoration is not permitted in the presence of nursery areas. | - | NC DMF |
| **Military Zones** | Location of military exclusion zones used for practice missions. | Exclusion | Excluded if a cell contained military protected area because habitat enhancement is not permitted in military zones due to the possibility of unexploded ordnance. | - | NC DEQ |
| **Navigational Channels** | Location of major navigational channels including the Intracoastal Waterway and Ferry routes. | Exclusion | Excluded if a cell contained the Intracoastal Waterway or Ferry routes to prevent navigational hazards to large vessels. | - | USGS |
