# Supplementary material for: Integrating ecosystem services considerations within a GIS-based habitat suitability index for oyster restoration
Source: PLoS One. 2019 Jan 25;14(1):e0210936. doi: 10.1371/journal.pone.0210936 (PMC6347164; doi:10.1371/journal.pone.0210936)
Supplement: S2 Table — Chlorophyll a concentrations were averaged across all pixels for a given sampling period to obtain a single value for a monthly mean chlorophyll a concentration and subsequently averaged across years within a given month (e.g., averaged across January 2003 through 2011). September, which corresponds with the fall phytoplankton bloom in Pamlico Sound, was determined to be the month of maximum average chlorophyll a concentration for the period of 2003 through 2011. (DOCX) [file pone.0210936.s002.docx]

| **Month** | **Mean Chlorophyll *a* Concentration ± SE (2003-2011; μg l^-1^)** |
| --- | --- |
| January | 21.63 ± 4.91 |
| February | 18.06 ± 5.49 |
| March | 17.76 ± 4.44 |
| April | 17.73 ± 2.71 |
| May | 20.21 ± 2.87 |
| June | 22.24 ± 3.03 |
| July | 22.88 ± 4.27 |
| August | 22.08 ± 3.02 |
| September | 22.92 ± 4.98 |
| October | 19.47 ± 1.96 |
| November | 16.92 ± 2.09 |
| December | 17.86 ± 3.71 |
