# Supplementary figures and images for: Integrating ecosystem services considerations within a GIS-based habitat suitability index for oyster restoration
Source: PLoS One. 2019 Jan 25;14(1):e0210936. doi: 10.1371/journal.pone.0210936 (PMC6347164; doi:10.1371/journal.pone.0210936)

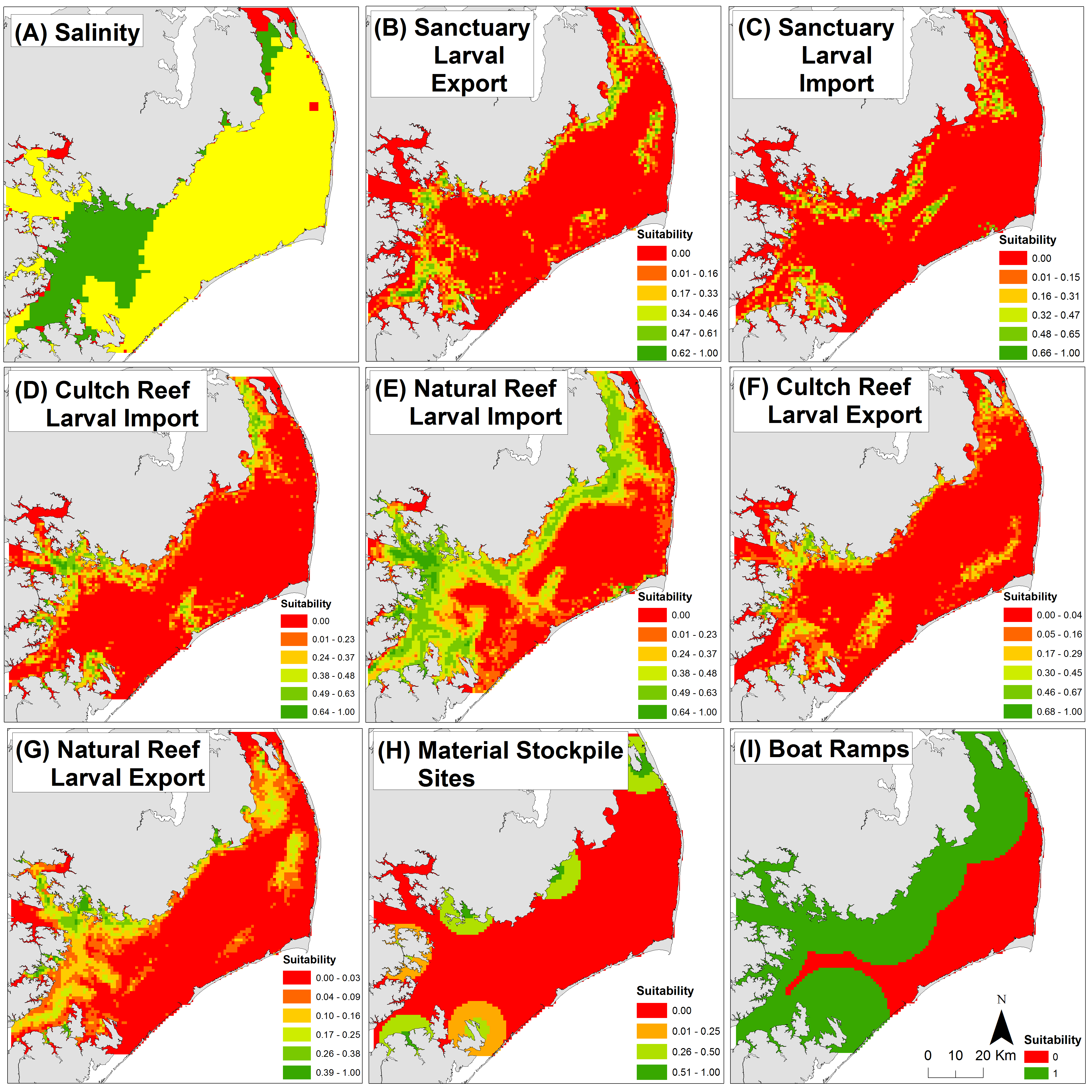

Supplement: S1 Fig — A) Salinity. B) Sanctuary larval export. C) Sanctuary larval import. D) Cultch reef larval import. E) Natural reef larval import. F) Cultch reef larval export. G) Natural reef larval export. H) Material stockpile sites. I) Boat ramps. Suitability increases from low (red) to high (green) for each layer. (TIF) [file pone.0210936.s003.tif]

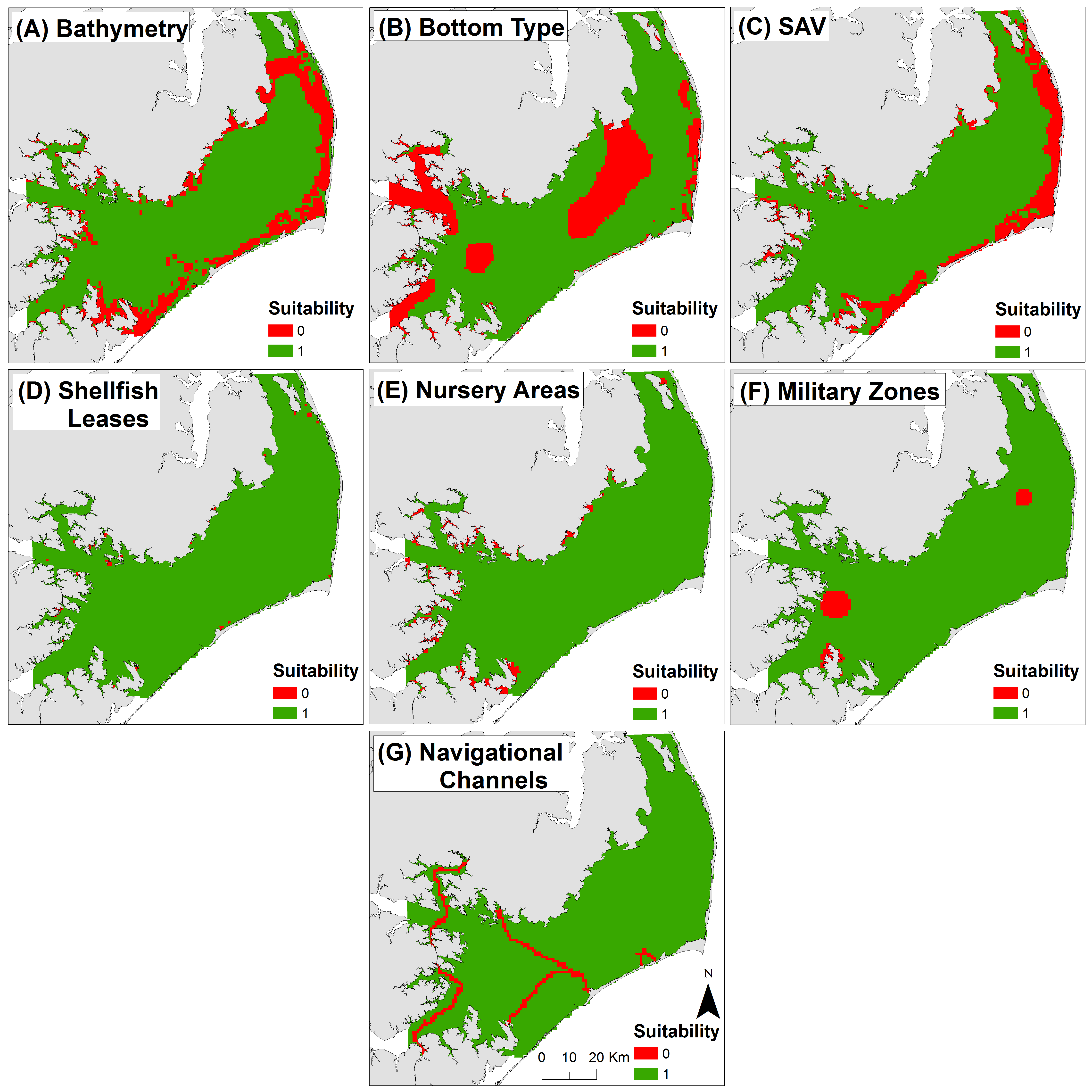

Supplement: S2 Fig — A) Bathymetry. B) Bottom Type. C) SAV. D) Shellfish leases. E) Nursery areas. F) Military zones. G) Navigational channels. Suitability increases from unsuitable (red) to optimal (green) for each layer. (TIF) [file pone.0210936.s004.tif]

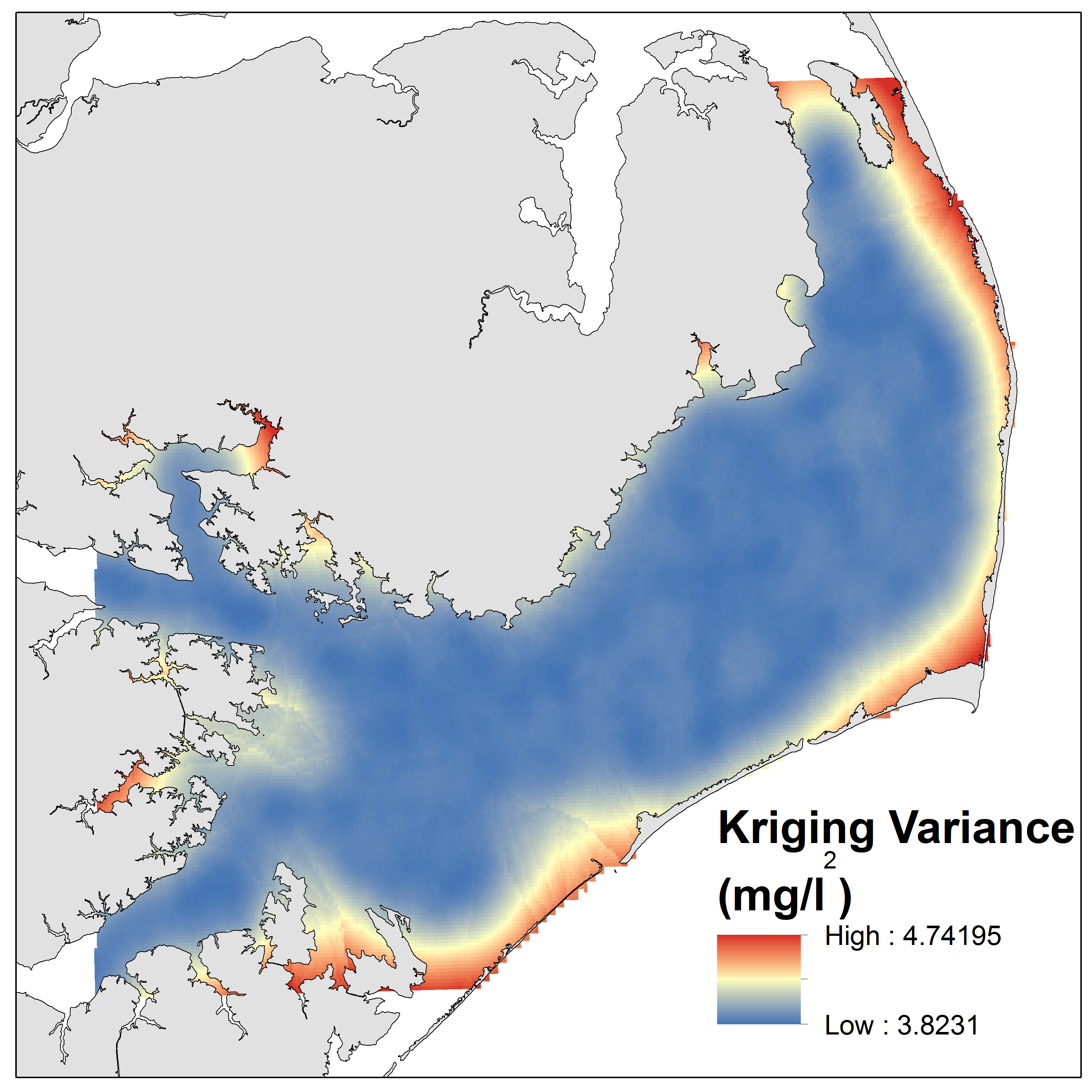

Supplement: S4 Fig — Fall sound-wide minimum benthic dissolved oxygen concentrations from 1996–2014 were interpolated using ordinary kriging to estimate minimum benthic dissolved oxygen throughout the system. (TIF) [file pone.0210936.s006.tif]

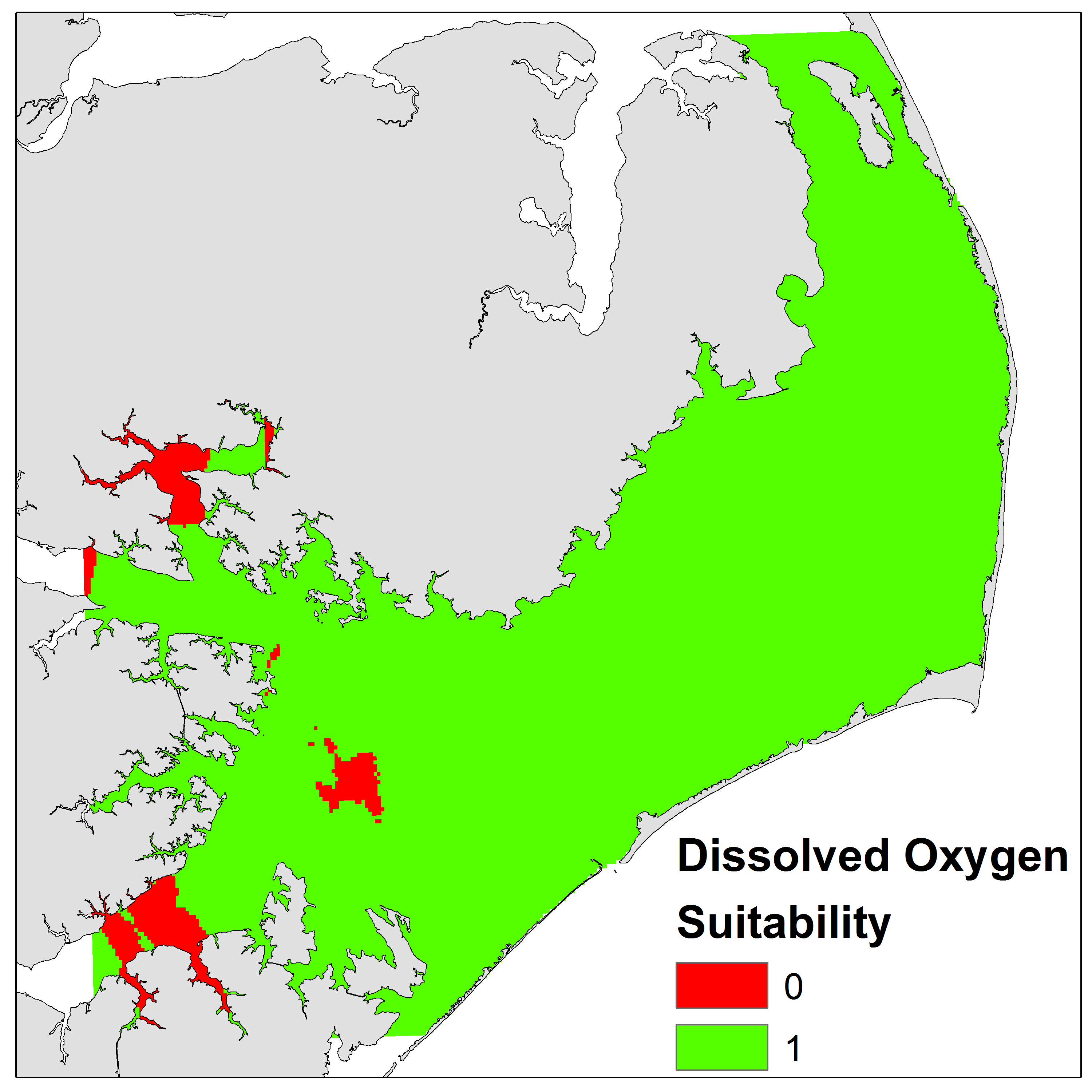

Supplement: S5 Fig — Note that this layer was not utilized within the analysis as it inappropriately considers areas of known episodic hypoxia (e.g., within the Neuse and Pamlico Rivers) as suitable. (TIF) [file pone.0210936.s007.tif]

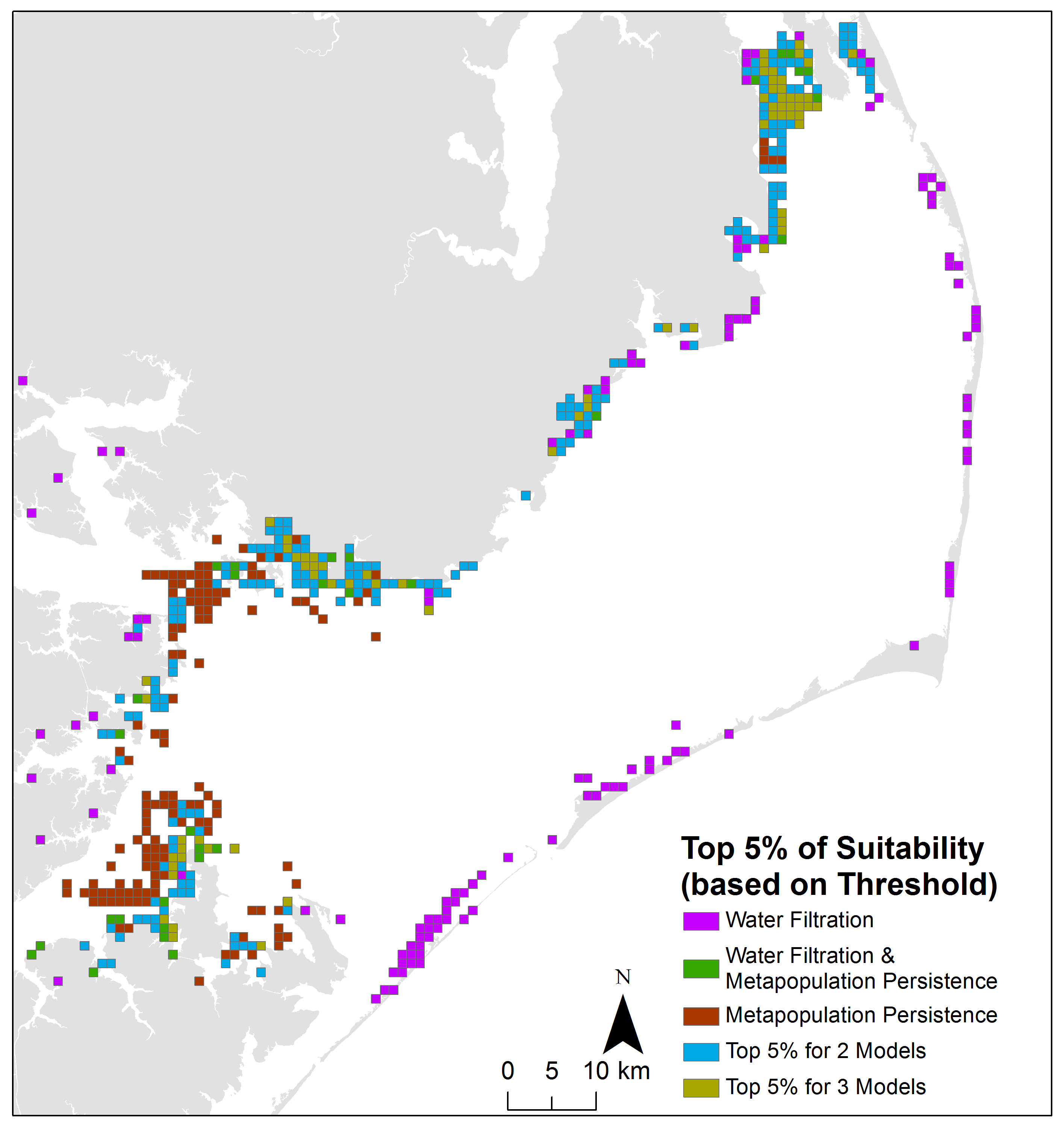

Supplement: S6 Fig — Locations that were identified within the top 5% for multiple HSI scenarios are indicated in blue (2 models) and gold (3 models). For example, ‘Top 5% for 2 Models’ may include areas identified within the top 5% for the ‘Water Filtration’ and the ‘Water Filtration & Metapopulation Persistence’ HSI scenarios, whereas ‘Top 5% for 3 Models’ includes areas identified within the top 5% for all three HSI scenarios. (TIF) [file pone.0210936.s008.tif]

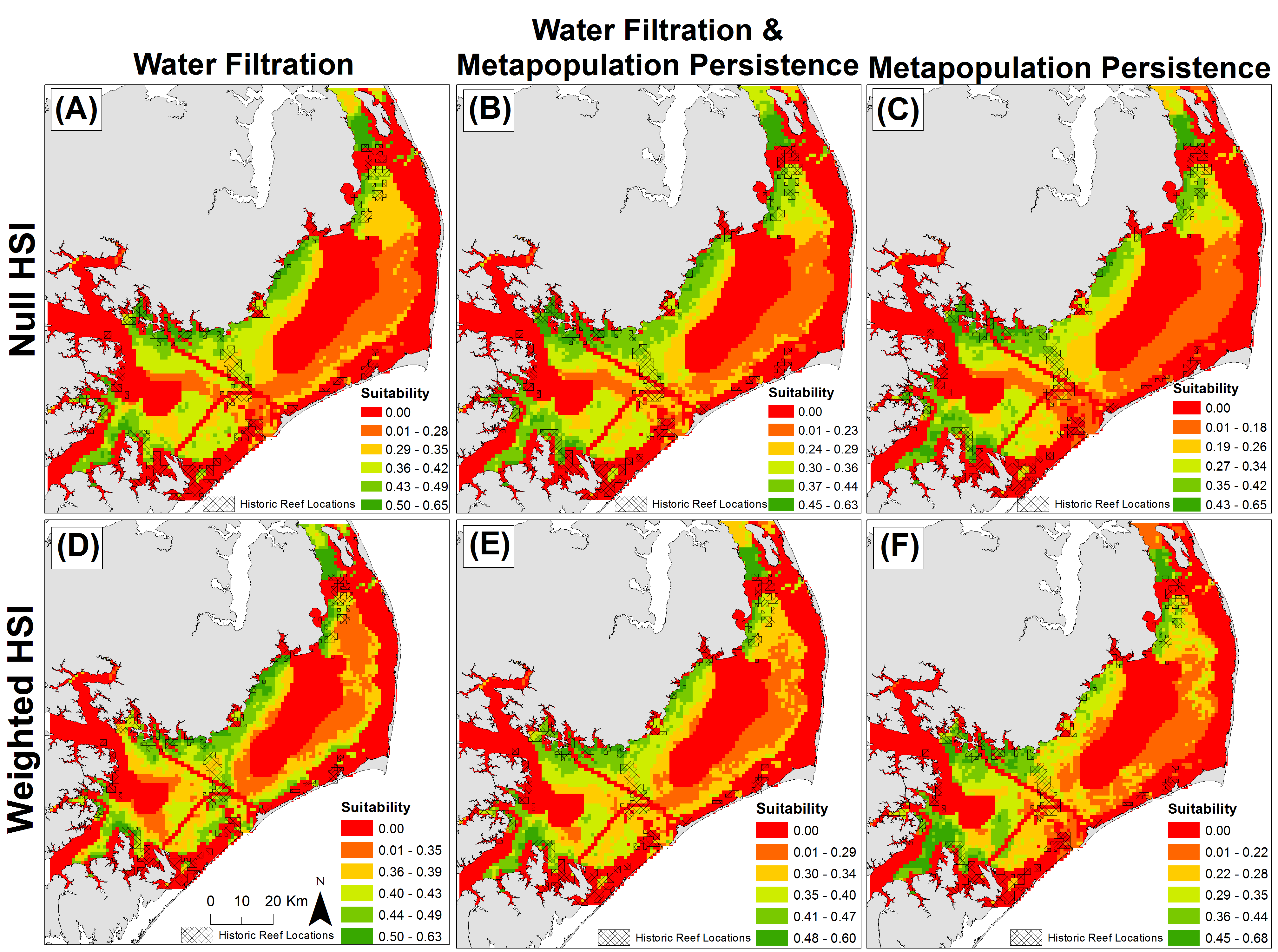

Supplement: S7 Fig — The location of historic oyster reefs are depicted by black crosshatch. (TIF) [file pone.0210936.s009.tif]
